# Supplementary material for: Cross-ancestral GWAS identifies 29 variants across head and neck cancer subsites
Source: Nat Commun. 2025 Oct 2;16:8787. doi: 10.1038/s41467-025-63842-z (PMC12491539; doi:10.1038/s41467-025-63842-z)
Supplement: Supplementary file 1 — Supplementary Information [file 41467_2025_63842_MOESM1_ESM.pdf]

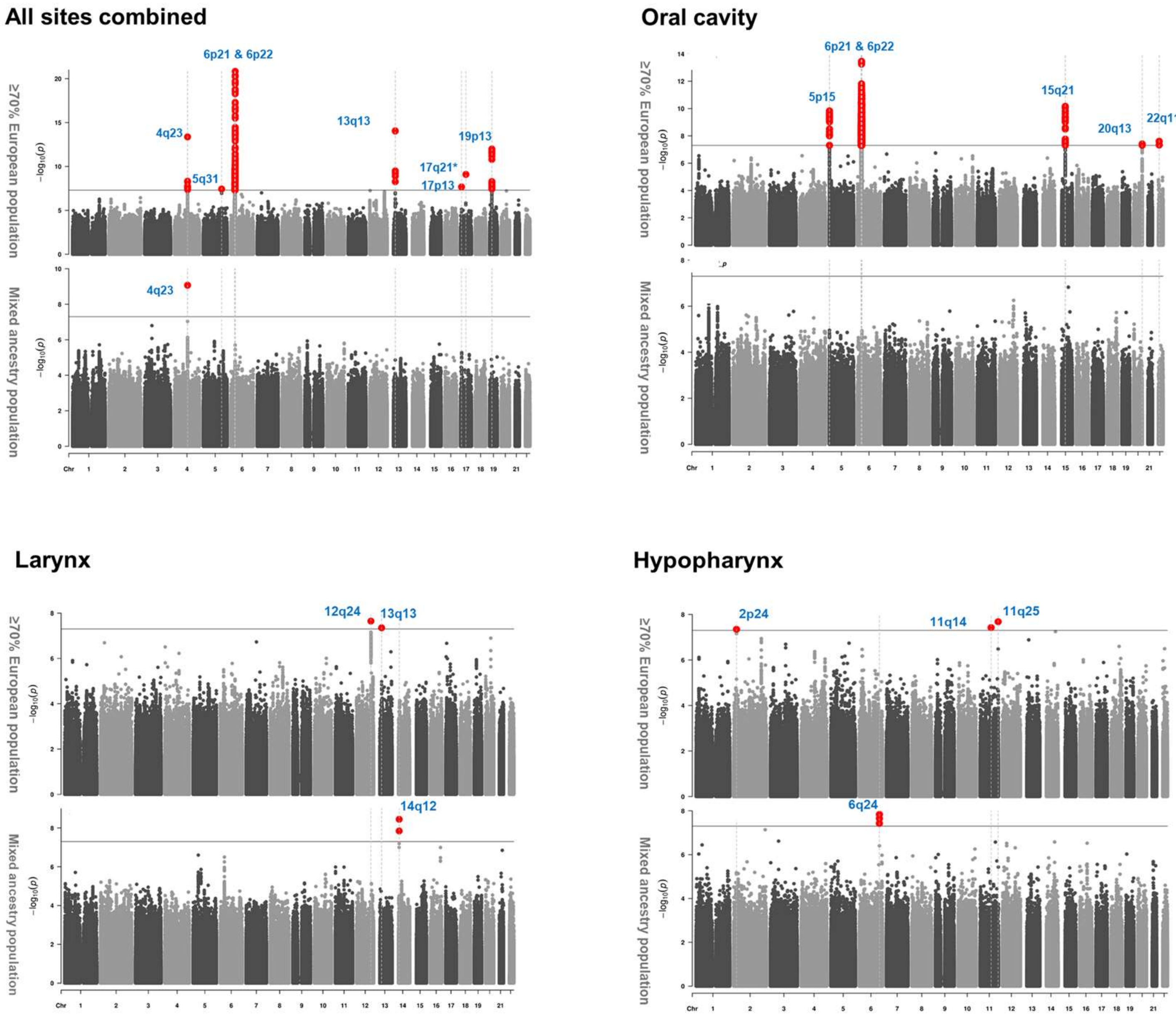

Supplementary Figure 1. Genome-wide association results for overall HNSCC, oral cavity, larynx and hypopharynx in meta-analysis of European and Mixed ancestry groups. The grey line indicates the genome-wide significance threshold of $5x10^{-8}$. Variants that met or exceeded this threshold are highlighted in red.

*In All sites combined the 17q21 variant was excluded due statistically significant heterogeneity across imputation batches/studies.

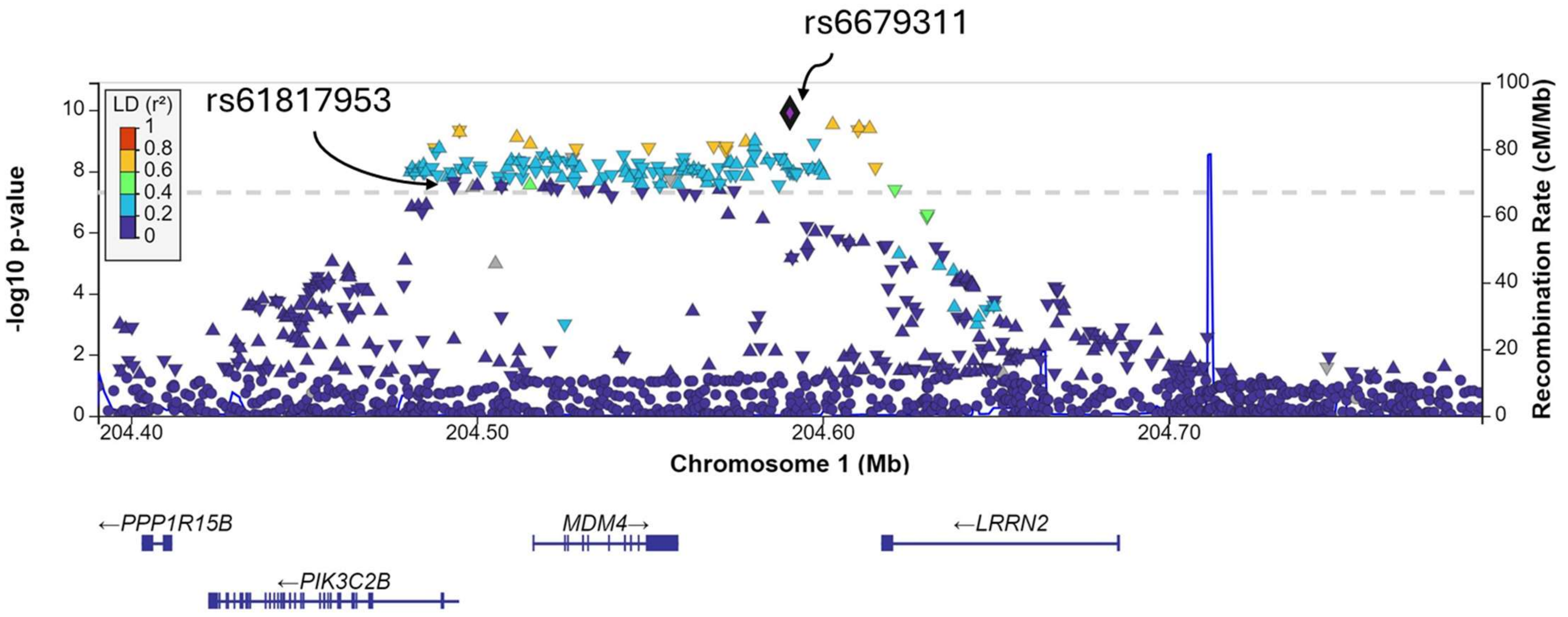

Supplementary Figure 2. Regional plot for two independent variants, rs61817953 and rs6679311, identified at 1q32. The x-axis represents the chromosome position, while the y-axis shows the –log10 P value. rs61817953, near *PIK3C2B* (OR (95%CI) = 0.90 (0.87, 0.93), p_{meta}= 2.17x10^{-8}) and rs6679311 near *MDM4,* a strong negative regulator of *TP53* (OR (95% CI) = 1.11 (1.07,1.14),p_{meta}=1.25x10^{-10}).

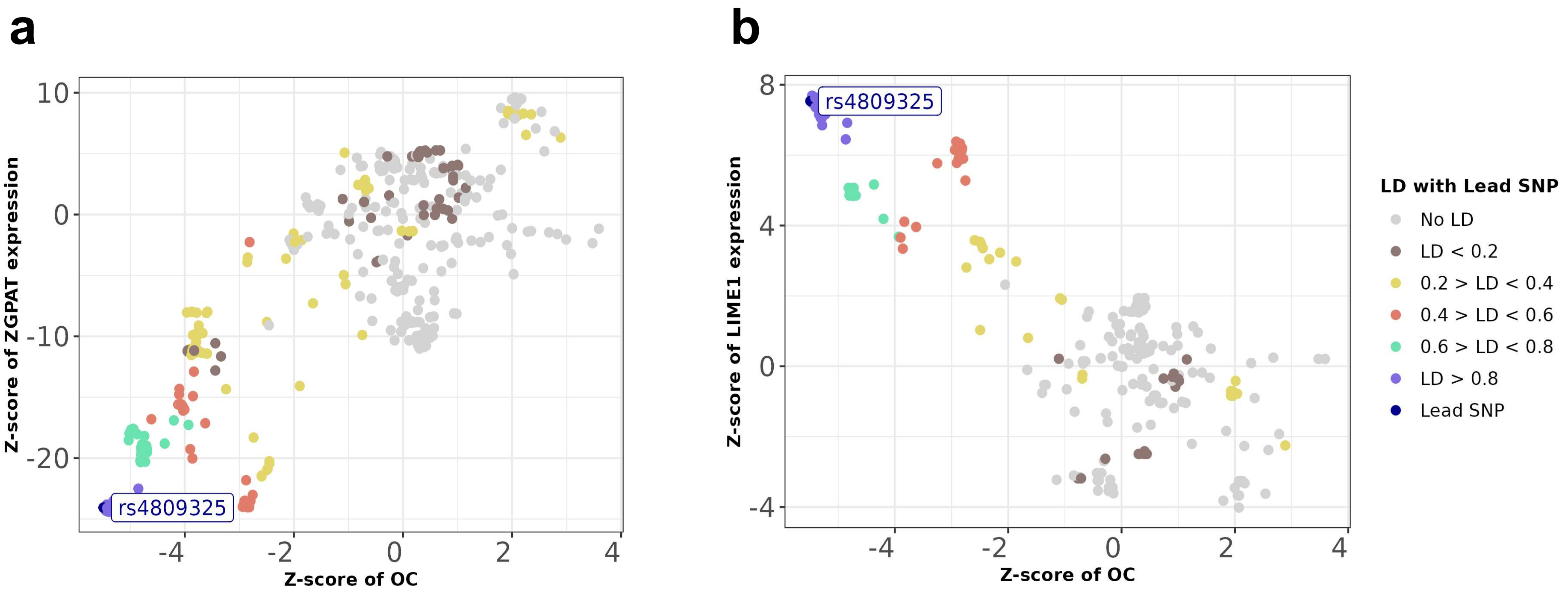

Supplementary Figure 3. Z-Z locus plot of rs4809325. a) rs4809325, colocalised with *ZGPAT* in whole blood (PP4 score= 0.97) and b) *LIME1* in esophagus mucosa (PP4 score=0.97); OC, Oral cavity.

a

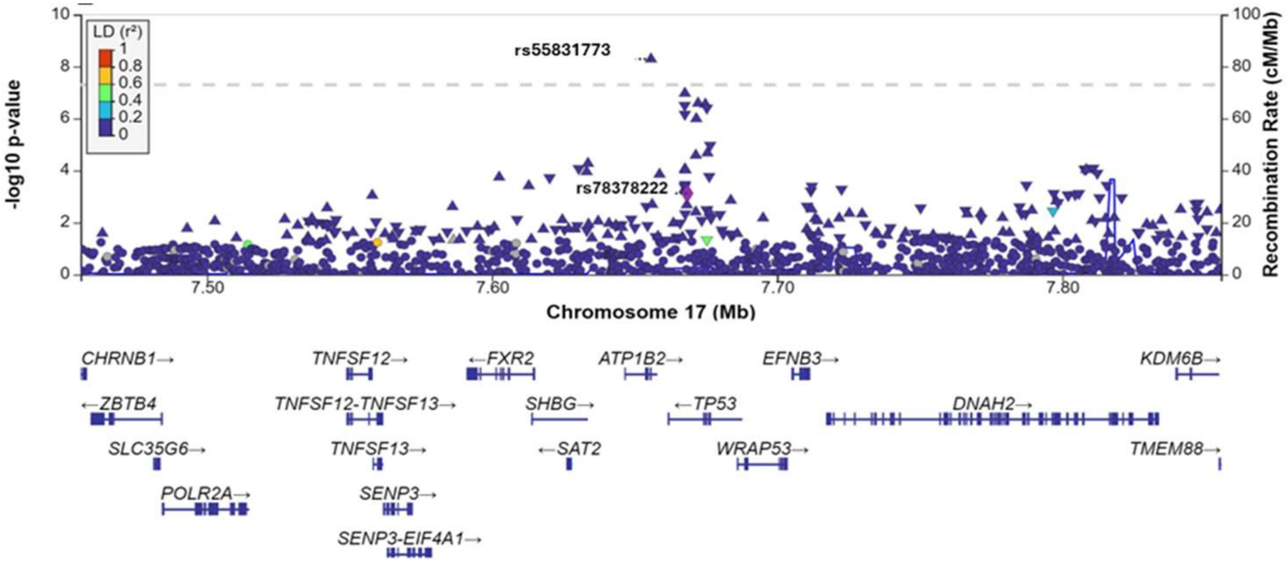

b

Subsite	p-value before conditioning	p-value after conditioning
**All sites combined (European ancestry group)**		
rs55831773	0.0004	0.001
rs78378222	2.16E-08	2.16E-08
**Larynx (European ancestry group)**		
rs55831773	2.16E-07	6.23E-07
rs78378222	0.0007	0.0007

c

Variable	NO.Case	NO.Control	OR.(95% Conf. Int.)	P-Heterogeneity
**Cross-ancestry meta-analysis**				
Larynx	4409	37722	1.21(1.13 to 1.29)	
**Genetic ancestry**				
European	2787	31833	1.23(1.14 to 1.32)	0.53
Mixed	1622	5889	1.17(1.05 to 1.31)	
**Sex**				
Men	3826	19246	1.22(1.14 to 1.31)	0.33
Women	583	18476	1.12(0.96 to 1.31)	
**Smoking Status**				
Never	552	15197	1.05(0.86 to 1.29)	0.21
Former	1726	9741	1.29(1.15 to 1.44)	
Current	2114	5675	1.26(1.12 to 1.41)	
**Drinking Status**				
Never	774	3398	1.13(0.96 to 1.33)	0.26
Ever	3366	26338	1.26(1.16 to 1.36)	
**Geographic Region**				
Europe	2003	30967	1.17(1.08 to 1.27)	0.7
North America	1354	3694	1.15(1.03 to 1.30)	
South America	739	2239	1.17(1.00 to 1.37)	
Middle East	282	504	1.00(0.79 to 1.28)	
**Subsite**				
Oral cavity	5596	37722	1.02(0.96 to 1.08)	0.97
Hypopharynx	898	37722	1.02(0.90 to 1.15)	
Oropharynx (HPV-)	1473	23132	1.00(0.91 to 1.11)	
Oropharynx (HPV+)	2212	22803	0.99(0.91 to 1.08)	

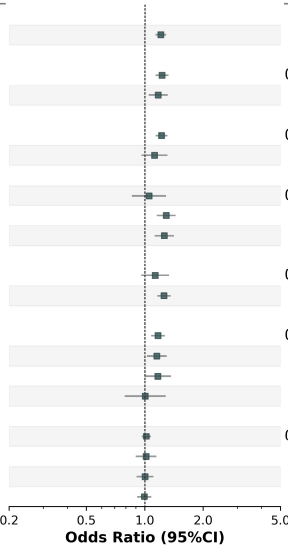

Supplementary Figure 4. Overview of the association signal at 17p13 for rs55831773. a) Regional association plot highlighting rs55831773, a splice polypyrimidine tract variant mapped to *ATP1B2*, associated with increased laryngeal cancer risk in the cross-ancestral analysis (OR (95% CI)=1.21 (1.13,1.29), $p_{meta}=5.1x10^{-9}$). b) *ATP1B2* is in close proximity to *TP53* but conditional analyses (performed in European population) confirm this variant is independent of rs78378222 (the rare *TP53* 3'-UTR variant) identified in overall HNSCC in European population. c) forest plot of odds ratio for rs55831773 variant stratified by sex, smoking- and drinking status, and geographic region within cross-ancestry laryngeal cancer meta-analysis. The risk-increasing effect of this variant was observed only in laryngeal cancer, with no effect on other subsites. In contrast, the *TP53* variant rs78378222 showed a protective effect in non-HPV-related HNSCC cancer sites.

Variable	NO.Case	NO.Control	OR.(95% Conf. Int.)	P-Heterogeneity
**Cross-ancestry meta-analysis**				
Larynx	4409	37722	1.25(1.00 to 1.55)	
**Genetic ancestry**				
European	2787	31833	0.82(0.63 to 1.07)	< 0.0001***
Mixed	1622	5889	3.38(2.26 to 5.07)	
**Sex**				
Men	3826	19246	1.22(0.96 to 1.54)	0.57
Women	583	18476	1.45(0.82 to 2.59)	
**Smoking Status**				
Never	552	15197	2.29(1.15 to 4.55)	0.08
Former	1726	9741	1.27(0.86 to 1.87)	
Current	2114	5675	0.93(0.62 to 1.38)	
**Drinking Status**				
Never	774	3398	2.71(1.53 to 4.79)	0.003**
Ever	3366	26338	1.05(0.79 to 1.38)	
**Geographic Region**				
Europe	2003	30967	1.17(0.89 to 1.52)	0.0009***
North America	1354	3694	1.00(0.68 to 1.48)	
South America	739	2239	3.05(1.91 to 4.87)	
Middle East	282	504	3.12(0.82 to 11.88)	
**Subsite**				
Oral cavity	5596	37723	1.04(0.86 to 1.26)	0.08
Hypopharynx	898	37689	0.61(0.36 to 1.03)	
Oropharynx (HPV-)	1473	23170	0.68(0.46 to 1.02)	
Oropharynx (HPV+)	2212	22803	0.83(0.62 to 1.10)	

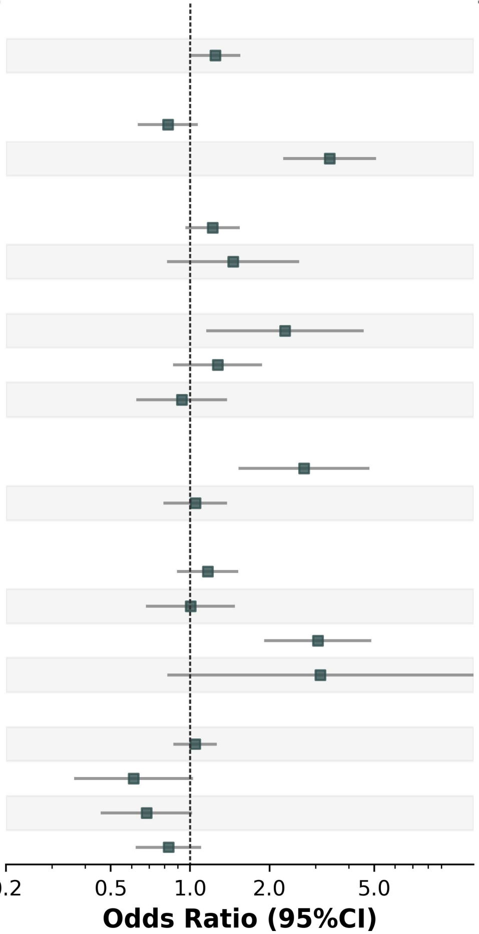

Supplementary Figure 5. Forest plot of odds ratios for rs200410709 showing significant heterogeneity in European-vs Mixed ancestry group. rs200410709, a deletion variant intronic within *STXBP6* (14q12), was linked to a large increased risk of LA (3.38 (2.26, 5.07), p=3.57x10^{-9}).

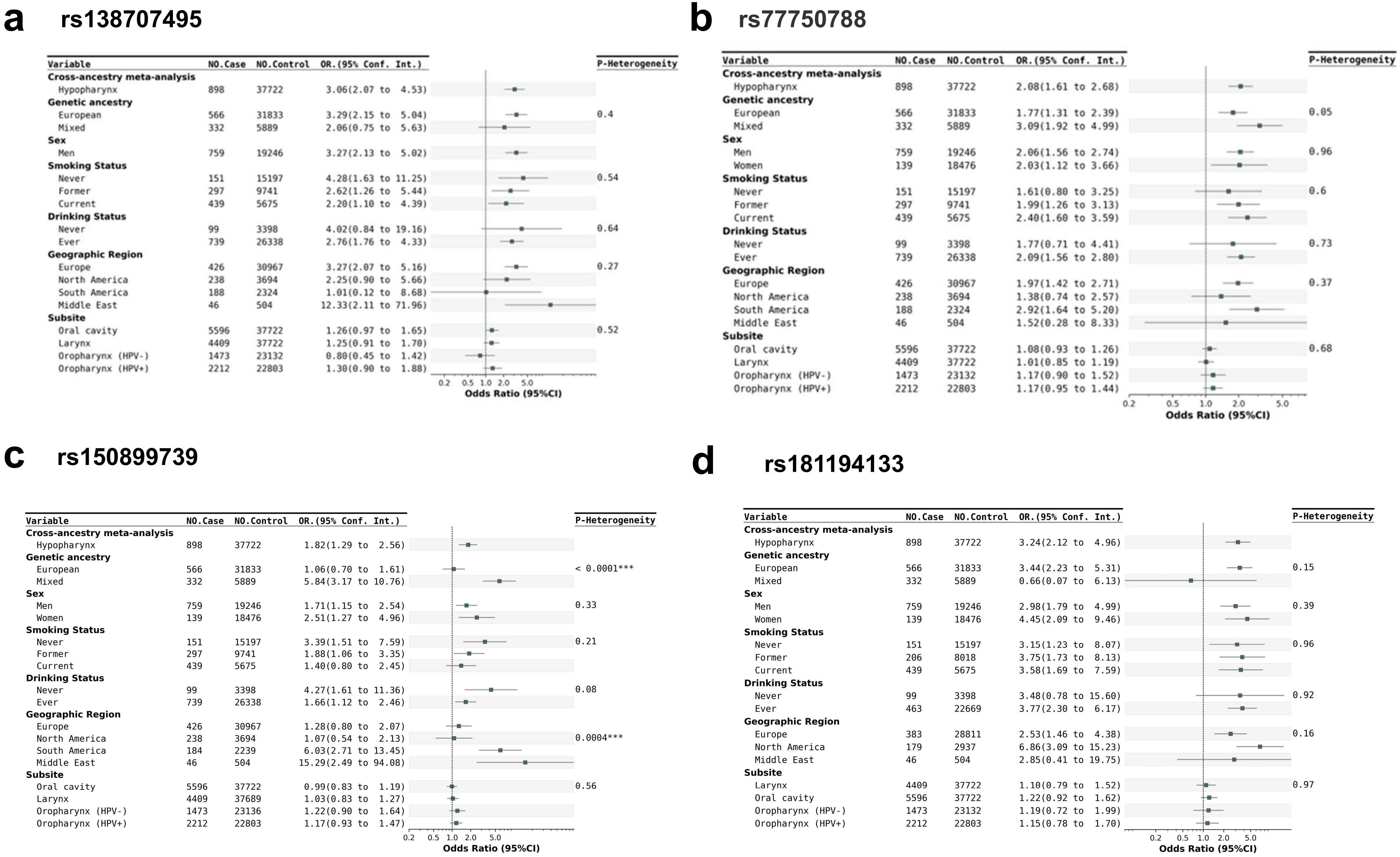
a
rs138707495
Variable NO.Case NO.Control OR.(95% Conf. Int.) P-Heterogeneity
Cross-ancestry meta-analysis
Hypopharynx 898 37722 3.06(2.07 to 4.53)
Genetic ancestry
European 566 31833 3.29(2.15 to 5.04) 0.4
Mixed 332 5889 2.06(0.75 to 5.63)
Sex
Men 759 19246 3.27(2.13 to 5.02)
Smoking Status
Never 151 15197 4.28(1.63 to 11.25) 0.54
Former 297 9741 2.62(1.26 to 5.44)
Current 439 5675 2.20(1.10 to 4.39)
Drinking Status
Never 99 3398 4.02(0.84 to 19.16) 0.64
Ever 739 26338 2.76(1.76 to 4.33)
Geographic Region
Europe 426 30967 3.27(2.07 to 5.16) 0.27
North America 238 3694 2.25(0.90 to 5.66)
South America 188 2324 1.01(0.12 to 8.68)
Middle East 46 504 12.33(2.11 to 71.96)
Subsite
Oral cavity 5596 37722 1.26(0.97 to 1.65) 0.52
Larynx 4409 37722 1.25(0.91 to 1.70)
Oropharynx (HPV-) 1473 23132 0.80(0.45 to 1.42)
Oropharynx (HPV+) 2212 22803 1.30(0.90 to 1.88)
0.2 0.5 1.0 2.0 5.0
Odds Ratio (95%CI)
b
rs77750788
Variable NO.Case NO.Control OR.(95% Conf. Int.) P-Heterogeneity
Cross-ancestry meta-analysis
Hypopharynx 898 37722 2.08(1.61 to 2.68)
Genetic ancestry
European 566 31833 1.77(1.31 to 2.39) 0.05
Mixed 332 5889 3.09(1.92 to 4.99)
Sex
Men 759 19246 2.06(1.56 to 2.74) 0.96
Women 139 18476 2.03(1.12 to 3.66)
Smoking Status
Never 151 15197 1.61(0.80 to 3.25) 0.6
Former 297 9741 1.99(1.26 to 3.13)
Current 439 5675 2.40(1.60 to 3.59)
Drinking Status
Never 99 3398 1.77(0.71 to 4.41) 0.73
Ever 739 26338 2.09(1.56 to 2.80)
Geographic Region
Europe 426 30967 1.97(1.42 to 2.71) 0.37
North America 238 3694 1.38(0.74 to 2.57)
South America 188 2324 2.92(1.64 to 5.20)
Middle East 46 504 1.52(0.28 to 8.33)
Subsite
Oral cavity 5596 37722 1.08(0.93 to 1.26) 0.68
Larynx 4409 37722 1.01(0.85 to 1.19)
Oropharynx (HPV-) 1473 23132 1.17(0.90 to 1.52)
Oropharynx (HPV+) 2212 22803 1.17(0.95 to 1.44)
0.2 0.5 1.0 2.0 5.0
Odds Ratio (95%CI)
c
rs150899739
Variable NO.Case NO.Control OR.(95% Conf. Int.) P-Heterogeneity
Cross-ancestry meta-analysis
Hypopharynx 898 37722 1.82(1.29 to 2.56)
Genetic ancestry
European 566 31833 1.06(0.70 to 1.61) < 0.0001***
Mixed 332 5889 5.84(3.17 to 10.76)
Sex
Men 759 19246 1.71(1.15 to 2.54) 0.33
Women 139 18476 2.51(1.27 to 4.96)
Smoking Status
Never 151 15197 3.39(1.51 to 7.59) 0.21
Former 297 9741 1.88(1.06 to 3.35)
Current 439 5675 1.40(0.80 to 2.45)
Drinking Status
Never 99 3398 4.27(1.61 to 11.36) 0.08
Ever 739 26338 1.66(1.12 to 2.46)
Geographic Region
Europe 426 30967 1.28(0.80 to 2.07)
North America 238 3694 1.07(0.54 to 2.13) 0.0004***
South America 184 2239 6.03(2.71 to 13.45)
Middle East 46 504 15.29(2.49 to 94.08)
Subsite
Oral cavity 5596 37722 0.99(0.83 to 1.19) 0.56
Larynx 4409 37689 1.03(0.83 to 1.27)
Oropharynx (HPV-) 1473 23136 1.22(0.90 to 1.64)
Oropharynx (HPV+) 2212 22803 1.17(0.93 to 1.47)
0.2 0.5 1.0 2.0 5.0
Odds Ratio (95%CI)
d
rs181194133
Variable NO.Case NO.Control OR.(95% Conf. Int.) P-Heterogeneity
Cross-ancestry meta-analysis
Hypopharynx 898 37722 3.24(2.12 to 4.96)
Genetic ancestry
European 566 31833 3.44(2.23 to 5.31) 0.15
Mixed 332 5889 0.66(0.07 to 6.13)
Sex
Men 759 19246 2.98(1.79 to 4.99) 0.39
Women 139 18476 4.45(2.09 to 9.46)
Smoking Status
Never 151 15197 3.15(1.23 to 8.07) 0.96
Former 206 8018 3.75(1.73 to 8.13)
Current 439 5675 3.58(1.69 to 7.59)
Drinking Status
Never 99 3398 3.48(0.78 to 15.60) 0.92
Ever 463 22669 3.77(2.30 to 6.17)
Geographic Region
Europe 383 28811 2.53(1.46 to 4.38) 0.16
North America 179 2937 6.86(3.09 to 15.23)
Middle East 46 504 2.85(0.41 to 19.75)
Subsite
Larynx 4409 37722 1.10(0.79 to 1.52) 0.97
Oral cavity 5596 37722 1.22(0.92 to 1.62)
Oropharynx (HPV-) 1473 23132 1.19(0.72 to 1.99)
Oropharynx (HPV+) 2212 22803 1.15(0.78 to 1.70)
0.2 0.5 1.0 2.0 5.0
Odds Ratio (95%CI)

e rs181777026

Variable	NO.Case	NO.Control	OR.(95% Conf. Int.)	P-Heterogeneity
**Cross-ancestry meta-analysis**				
Hypopharynx	898	37722	2.51(1.80 to 3.50)	
**Genetic ancestry**				
European	566	31833	2.81(1.94 to 4.05)	0.17
Mixed	332	5889	1.56(0.72 to 3.34)	
**Sex**				
Men	759	19246	2.40(1.65 to 3.48)	0.37
Women	100	16715	3.48(1.67 to 7.22)	
**Smoking Status**				
Never	102	12587	1.87(0.61 to 5.77)	0.25
Former	297	9741	3.26(1.91 to 5.56)	
Current	439	5675	1.72(0.97 to 3.04)	
**Drinking Status**				
Never	99	3398	1.06(0.19 to 5.72)	0.31
Ever	739	26338	2.58(1.79 to 3.74)	
**Geographic Region**				
Europe	426	30967	2.31(1.50 to 3.55)	0.43
North America	238	3694	3.35(1.79 to 6.25)	
South America	184	2239	1.66(0.63 to 4.33)	
**Subsite**				
Oral cavity	5596	37722	1.35(1.10 to 1.65)	0.55
Larynx	4409	37722	1.07(0.84 to 1.38)	
Oropharynx (HPV-)	1473	23132	1.21(0.82 to 1.78)	
Oropharynx (HPV+)	2212	22803	1.13(0.82 to 1.55)	

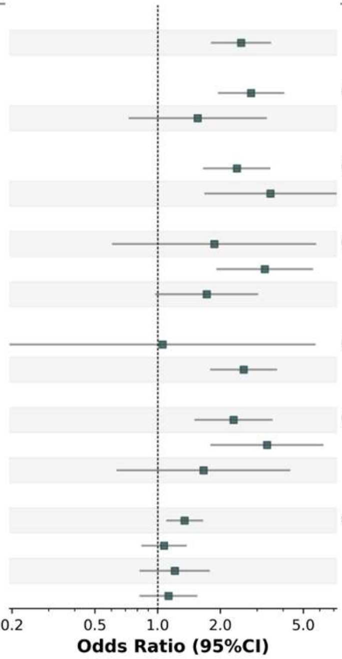

Supplementary Figure 6. Forest plots of odds ratios for five variants identified in hypopharyngeal cancer (HPC). a) rs138707495 located in the 3' UTR of *GDF7* at the locus 2p24 identified in meta-analysis. b) rs77750788 near *IGSF9B* at the locus of 11q25. c) rs150899739 in *SASH1* at locus 6q24. d) rs181194133 an intronic variant in *OPCML* at the locus 11q25. e) rs181777026 intergenic variant near *TENM4* at the locus 11q14.

a Cross-ancestry (all sites combined)

rs61817953

Variable	NO.Case	NO.Control	OR.(95% Conf. Int.)	P-Heterogeneity
**Cross-ancestry meta-analysis**				
All sites combined	19073	38857	0.90(0.87 to 0.93)	
**Genetic ancestry**				
European	13554	32914	0.91(0.87 to 0.94)	0.51
Mixed	5519	5943	0.88(0.81 to 0.95)	
**Sex**				
Men	14848	20074	0.91(0.87 to 0.95)	0.67
Women	4225	18783	0.89(0.83 to 0.96)	
**Smoking Status**				
Never	3827	15197	0.90(0.84 to 0.98)	0.9
Former	5811	9741	0.90(0.83 to 0.96)	
Current	6944	5675	0.92(0.85 to 1.00)	
**Drinking Status**				
Never	2983	3398	0.93(0.83 to 1.03)	0.71
Ever	12866	26338	0.91(0.86 to 0.95)	
**Geographic Region**				
Europe	7808	30967	0.91(0.87 to 0.95)	0.93
North America	7555	4829	0.89(0.84 to 0.96)	
South America	3030	2324	0.90(0.80 to 1.02)	
Middle East	505	504	0.91(0.73 to 1.14)	
South Asia	175	233	1.05(0.74 to 1.48)	
**Subsite**				
Oral cavity	5596	37722	0.92(0.87 to 0.97)	0.9
Larynx	4409	37722	0.88(0.83 to 0.95)	
Hypopharynx	566	31833	0.88(0.75 to 1.02)	
Oropharynx (HPV-)	1473	23132	0.91(0.82 to 1.01)	
Oropharynx (HPV+)	2212	22803	0.89(0.81 to 0.97)	

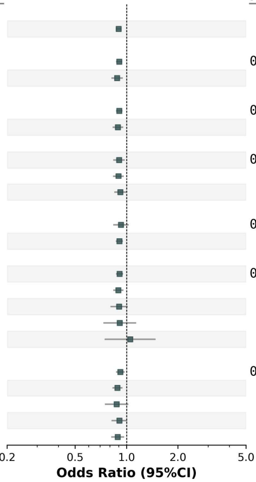

rs6679311

Variable	NO.Case	NO.Control	OR.(95% Conf. Int.)	P-Heterogeneity
**Cross-ancestry meta-analysis**				
All sites combined	19073	38857	1.11(1.07 to 1.14)	
**Genetic ancestry**				
European	13554	32914	1.10(1.06 to 1.14)	0.3
Mixed	5519	5943	1.14(1.07 to 1.21)	
**Sex**				
Men	14848	20074	1.10(1.07 to 1.15)	0.83
Women	4225	18783	1.11(1.05 to 1.18)	
**Smoking Status**				
Never	3827	15197	1.11(1.04 to 1.18)	0.46
Former	5811	9741	1.12(1.06 to 1.19)	
Current	6944	5675	1.06(0.99 to 1.14)	
**Drinking Status**				
Never	2983	3398	1.09(1.00 to 1.19)	0.97
Ever	12866	26338	1.09(1.05 to 1.13)	
**Geographic Region**				
Europe	7808	30967	1.06(1.02 to 1.11)	0.02*
North America	7555	4829	1.18(1.11 to 1.25)	
South America	3030	2324	1.14(1.05 to 1.24)	
Middle East	505	504	1.07(0.88 to 1.31)	
South Asia	175	233	0.83(0.60 to 1.16)	
**Subsite**				
Oral cavity	5596	37722	1.09(1.04 to 1.15)	0.42
Larynx	4409	37722	1.11(1.05 to 1.17)	
Hypopharynx	898	37722	1.22(1.10 to 1.35)	
Oropharynx (HPV-)	1473	23132	1.13(1.04 to 1.23)	
Oropharynx (HPV+)	2212	22803	1.13(1.05 to 1.21)	

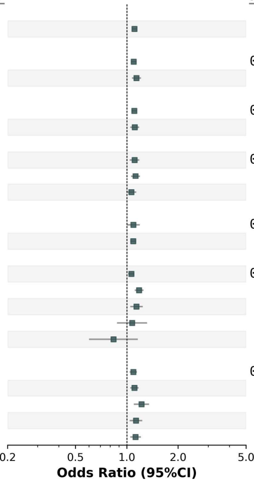

rs17529509

Variable	NO.Case	NO.Control	OR.(95% Conf. Int.)	P-Heterogeneity
**Cross-ancestry meta-analysis**				
All sites combined	19073	38857	0.83(0.79 to 0.87)	
**Genetic ancestry**				
European	13554	32914	0.85(0.80 to 0.90)	0.13
Mixed	5519	5943	0.78(0.70 to 0.86)	
**Sex**				
Men	14848	20074	0.81(0.76 to 0.86)	0.04*
Women	4225	18783	0.90(0.82 to 0.99)	
**Smoking Status**				
Never	3827	15197	0.95(0.86 to 1.05)	0.04*
Former	5811	9741	0.82(0.74 to 0.90)	
Current	6944	5675	0.81(0.73 to 0.90)	
**Drinking Status**				
Never	2983	3398	0.89(0.78 to 1.02)	0.36
Ever	12866	26338	0.83(0.78 to 0.89)	
**Geographic Region**				
Europe	7808	30967	0.84(0.79 to 0.90)	0.21
North America	7555	4829	0.87(0.80 to 0.95)	
South America	3030	2324	0.72(0.62 to 0.84)	
Middle East	505	504	0.81(0.61 to 1.07)	
South Asia	175	233	1.33(0.59 to 3.01)	
**Subsite**				
Oral cavity	5596	37722	0.84(0.78 to 0.91)	0.01*
Larynx	4409	37722	0.86(0.79 to 0.94)	
Hypopharynx	2898	37722	0.63(0.51 to 0.77)	
Oropharynx (HPV-)	1473	23132	0.80(0.69 to 0.93)	
Oropharynx (HPV+)	2212	22803	0.93(0.83 to 1.04)	

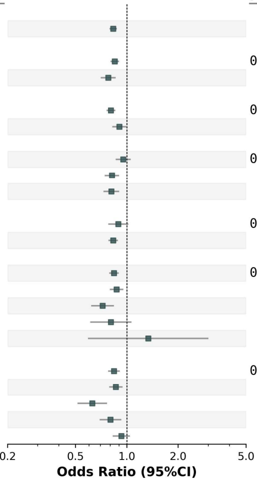

rs58223772

Variable	NO.Case	NO.Control	OR.(95% Conf. Int.)	P-Heterogeneity
**Cross-ancestry meta-analysis**				
All sites combined	19073	38857	0.90(0.87 to 0.93)	
**Genetic ancestry**				
European	13554	32914	0.92(0.88 to 0.95)	0.17
Mixed	5519	5943	0.87(0.82 to 0.93)	
**Sex**				
Men	14848	20074	0.89(0.86 to 0.93)	0.25
Women	4225	18783	0.93(0.87 to 0.99)	
**Smoking Status**				
Never	3827	15197	0.93(0.87 to 1.00)	0.03*
Former	5811	9741	0.95(0.89 to 1.01)	
Current	6944	5675	0.84(0.79 to 0.91)	
**Drinking Status**				
Never	2983	3398	0.94(0.86 to 1.03)	0.59
Ever	12866	26338	0.91(0.87 to 0.95)	
**Geographic Region**				
Europe	7808	30967	0.93(0.89 to 0.98)	0.08
North America	7555	4829	0.90(0.85 to 0.96)	
South America	3030	2324	0.81(0.74 to 0.89)	
Middle East	505	504	0.94(0.77 to 1.14)	
South Asia	175	233	0.98(0.73 to 1.32)	
**Subsite**				
Oral cavity	3511	31833	0.86(0.80 to 0.92)	0.0008***
Larynx	4409	37722	0.94(0.88 to 1.00)	
Hypopharynx	898	37722	0.87(0.78 to 0.97)	
Oropharynx (HPV-)	1473	23132	0.77(0.70 to 0.85)	
Oropharynx (HPV+)	2212	22803	0.98(0.91 to 1.06)	

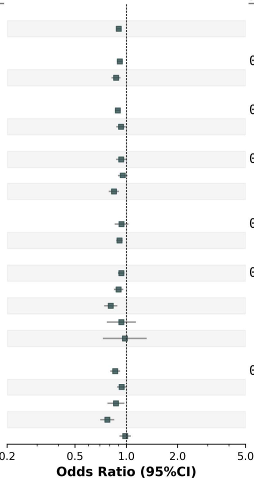

a Cross-ancestry (all sites combined)

rs1131769

Variable	NO.Case	NO.Control	OR.(95% Conf. Int.)	P-Heterogeneity
**Cross-ancestry meta-analysis**				
All sites combined	19073	38857	1.13(1.09 to 1.18)	
**Genetic ancestry**				
European	13554	32914	1.15(1.09 to 1.20)	0.45
Mixed	5519	5943	1.10(1.02 to 1.20)	
**Sex**				
Men	14848	20074	1.13(1.08 to 1.19)	0.68
Women	4225	18783	1.11(1.03 to 1.20)	
**Smoking Status**				
Never	3827	15197	1.10(1.01 to 1.20)	0.67
Former	5811	9741	1.16(1.07 to 1.26)	
Current	6944	5675	1.14(1.05 to 1.25)	
**Drinking Status**				
Never	2983	3398	1.18(1.05 to 1.33)	0.83
Ever	12866	26338	1.16(1.10 to 1.22)	
**Geographic Region**				
Europe	7808	30967	1.16(1.10 to 1.22)	0.83
North America	7555	4829	1.12(1.04 to 1.21)	
South America	3030	2324	1.08(0.97 to 1.21)	
Middle East	505	504	1.21(0.93 to 1.57)	
South Asia	175	233	1.15(0.70 to 1.89)	
**Subsite**				
Oral cavity	5596	37690	1.22(1.15 to 1.30)	0.001**
Larynx	4409	37722	1.15(1.07 to 1.23)	
Hypopharynx	898	37689	1.15(1.00 to 1.32)	
Oropharynx (HPV-)	1473	23132	1.13(1.01 to 1.26)	
Oropharynx (HPV+)	2212	22803	0.94(0.85 to 1.04)	

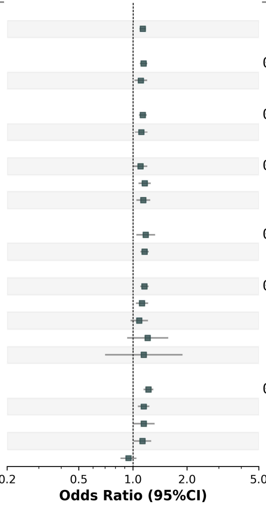

rs541752611

Variable	NO.Case	NO.Control	OR.(95% Conf. Int.)	P-Heterogeneity
**Cross-ancestry meta-analysis**				
All sites combined	19073	38857	1.22(1.14 to 1.31)	
**Genetic ancestry**				
European	13554	32914	1.26(1.16 to 1.36)	0.15
Mixed	5519	5943	1.12(0.98 to 1.28)	
**Sex**				
Men	14848	20074	1.25(1.15 to 1.36)	0.32
Women	4225	18783	1.16(1.01 to 1.32)	
**Smoking Status**				
Never	3827	15197	1.21(1.06 to 1.39)	0.23
Former	5811	9741	1.26(1.11 to 1.43)	
Current	6944	5675	1.06(0.91 to 1.24)	
**Drinking Status**				
Never	2983	3398	1.06(0.86 to 1.32)	0.5
Ever	12866	26338	1.15(1.05 to 1.25)	
**Geographic Region**				
Europe	7808	30967	1.23(1.13 to 1.34)	0.59
North America	7555	4829	1.21(1.07 to 1.38)	
South America	3030	2324	1.12(0.95 to 1.33)	
Middle East	505	504	0.70(0.32 to 1.52)	
South Asia	175	233	1.09(0.16 to 7.36)	
**Subsite**				
Oral cavity	5596	37690	1.13(1.01 to 1.25)	0.0001***
Larynx	4409	37722	1.07(0.94 to 1.21)	
Hypopharynx	898	37689	1.24(0.99 to 1.55)	
Oropharynx (HPV-)	1473	23132	1.19(0.99 to 1.43)	
Oropharynx (HPV+)	2212	22803	1.64(1.42 to 1.89)	

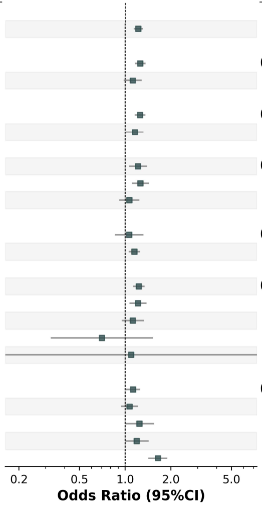

rs9266806

Variable	NO.Case	NO.Control	OR.(95% Conf. Int.)	P-Heterogeneity
**Cross-ancestry meta-analysis**				
All sites combined	19073	38857	0.89(0.86 to 0.92)	
**Genetic ancestry**				
European	13554	32914	0.87(0.84 to 0.91)	0.1
Mixed	5519	5943	0.93(0.87 to 0.99)	
**Sex**				
Men	14848	20074	0.88(0.85 to 0.92)	0.49
Women	4225	18783	0.91(0.85 to 0.97)	
**Smoking Status**				
Never	3827	15197	0.87(0.81 to 0.94)	0.37
Former	5811	9741	0.90(0.84 to 0.96)	
Current	6944	5675	0.94(0.87 to 1.01)	
**Drinking Status**				
Never	2983	3398	0.99(0.90 to 1.09)	0.12
Ever	12866	26338	0.91(0.87 to 0.95)	
**Geographic Region**				
Europe	7808	30967	0.89(0.85 to 0.93)	0.43
North America	7555	4829	0.88(0.82 to 0.94)	
South America	3030	2324	0.95(0.87 to 1.05)	
Middle East	505	504	0.97(0.80 to 1.19)	
South Asia	175	233	1.02(0.75 to 1.40)	
**Subsite**				
Oral cavity	5595	37690	0.93(0.88 to 0.98)	< 0.0001***
Larynx	4409	37722	0.97(0.92 to 1.04)	
Hypopharynx	898	37689	0.88(0.78 to 0.99)	
Oropharynx (HPV-)	1473	23132	0.93(0.85 to 1.02)	
Orophraynx (HPV+)	2212	22803	0.71(0.65 to 0.77)	

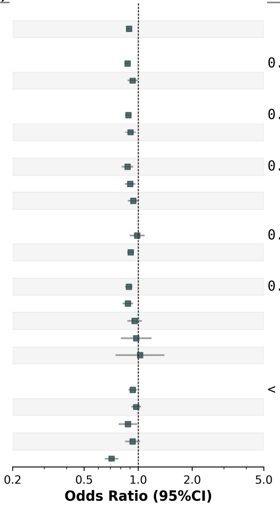

rs9282195

Variable	NO.Case	NO.Control	OR.(95% Conf. Int.)	P-Heterogeneity
**Cross-ancestry meta-analysis**				
All sites combined	19073	38857	0.84(0.81 to 0.87)	
**Genetic ancestry**				
European	13554	32914	0.83(0.80 to 0.86)	0.42
Mixed	5519	5943	0.86(0.80 to 0.93)	
**Sex**				
Men	14848	20074	0.83(0.79 to 0.86)	0.15
Women	4225	18783	0.87(0.82 to 0.93)	
**Smoking Status**				
Never	3827	15197	0.85(0.79 to 0.91)	0.46
Former	5811	9741	0.84(0.79 to 0.90)	
Current	6944	5675	0.89(0.83 to 0.96)	
**Drinking Status**				
Never	2983	3398	0.87(0.79 to 0.97)	0.58
Ever	12866	26338	0.84(0.81 to 0.88)	
**Geographic Region**				
Europe	7808	30967	0.85(0.81 to 0.89)	0.53
North America	7555	4829	0.82(0.77 to 0.88)	
South America	3030	2324	0.84(0.76 to 0.93)	
Middle East	505	504	0.76(0.56 to 1.02)	
South Asia	175	233	1.13(0.76 to 1.69)	
**Subsite**				
Oral cavity	5596	37722	0.84(0.79 to 0.88)	< .0001***
Larynx	4409	37722	0.94(0.88 to 1.00)	
Hypopharynx	898	37722	0.83(0.73 to 0.94)	
Oropharynx (HPV-)	1473	23132	0.85(0.77 to 0.94)	
Oropharynx (HPV+)	2212	22803	0.69(0.63 to 0.75)	

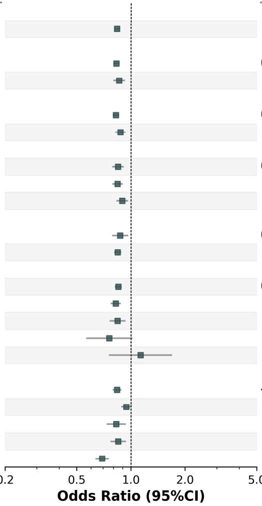

a **Cross-ancestry (all sites combined)**

rs13215307

Variable	NO.Case	NO.Control	OR.(95% Conf. Int.)	P-Heterogeneity
**Cross-ancestry meta-analysis**				
All sites combined	19073	38857	0.83(0.77 to 0.88)	
**Genetic ancestry**				
European	13554	32914	0.81(0.75 to 0.87)	0.23
Mixed	5519	5943	0.88(0.78 to 1.00)	
**Sex**				
Men	14848	20074	0.82(0.76 to 0.88)	0.48
Women	4225	18783	0.87(0.76 to 0.99)	
**Smoking Status**				
Never	3827	15197	0.84(0.73 to 0.97)	0.99
Former	5811	9741	0.83(0.74 to 0.95)	
Current	6944	5675	0.84(0.73 to 0.95)	
**Drinking Status**				
Never	2983	3398	0.93(0.77 to 1.12)	0.24
Ever	12866	26338	0.82(0.75 to 0.89)	
**Geographic Region**				
Europe	7808	30967	0.87(0.80 to 0.94)	0.98
North America	7555	4829	0.83(0.73 to 0.94)	
South America	3030	2324	0.88(0.73 to 1.05)	
Middle East	505	504	0.88(0.61 to 1.26)	
South Asia	175	233	0.92(0.15 to 5.67)	
**Subsite**				
Oral cavity	5596	37723	0.82(0.74 to 0.91)	0.4
Larynx	4409	37722	0.91(0.81 to 1.02)	
Hypopharynx	898	37722	0.81(0.64 to 1.02)	
Oropharynx (HPV-)	1473	23132	0.73(0.60 to 0.89)	
Oropharynx (HPV+)	2212	22803	0.81(0.69 to 0.95)	

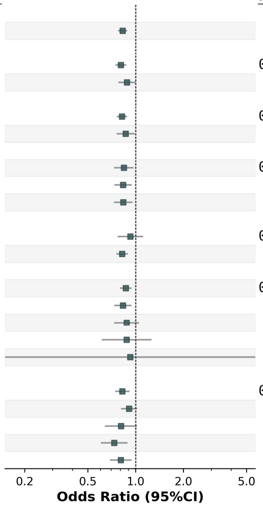

rs12314527

Variable	NO.Case	NO.Control	OR.(95% Conf. Int.)	P-Heterogeneity
**Cross-ancestry meta-analysis**				
All sites combined	19073	38857	1.11(1.07 to 1.15)	
**Genetic ancestry**				
European	13554	32914	1.12(1.07 to 1.17)	0.5
Mixed	5519	5943	1.09(1.02 to 1.16)	
**Sex**				
Men	14848	20074	1.12(1.07 to 1.17)	0.52
Women	4225	18783	1.09(1.02 to 1.17)	
**Smoking Status**				
Never	3827	15197	1.02(0.94 to 1.10)	0.03*
Former	5811	9741	1.12(1.05 to 1.20)	
Current	6944	5675	1.17(1.09 to 1.26)	
**Drinking Status**				
Never	2983	3398	1.05(0.95 to 1.16)	0.16
Ever	12866	26338	1.13(1.08 to 1.18)	
**Geographic Region**				
Europe	7808	30967	1.11(1.06 to 1.17)	0.74
North America	7555	4829	1.12(1.05 to 1.20)	
South America	3030	2324	1.04(0.95 to 1.14)	
Middle East	505	504	1.13(0.91 to 1.41)	
South Asia	175	233	1.11(0.79 to 1.58)	
**Subsite**				
Oral cavity	5596	37723	1.05(1.00 to 1.11)	0.0001***
Larynx	4409	37722	1.20(1.13 to 1.28)	
Hypopharynx	898	37689	1.17(1.04 to 1.32)	
Oropharynx (HPV-)	1473	23132	1.24(1.13 to 1.36)	
Oropharynx (HPV+)	2212	22803	0.98(0.90 to 1.07)	

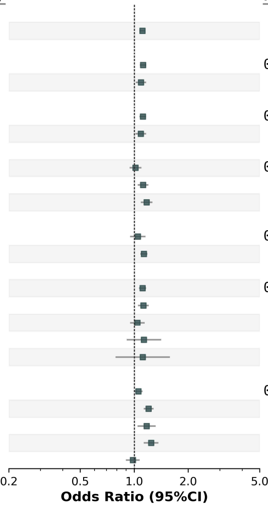

rs7334543

Variable	NO.Case	NO.Control	OR.(95% Conf. Int.)	P-Heterogeneity
**Cross-ancestry meta-analysis**				
All sites combined	19073	38857	0.91(0.88 to 0.94)	
**Genetic ancestry**				
European	13554	32914	0.91(0.88 to 0.95)	0.98
Mixed	5519	5943	0.91(0.85 to 0.97)	
**Sex**				
Men	14848	20074	0.91(0.87 to 0.94)	0.46
Women	4225	18783	0.93(0.88 to 0.99)	
**Smoking Status**				
Never	3827	15197	0.90(0.84 to 0.97)	0.78
Former	5811	9741	0.93(0.88 to 0.99)	
Current	6944	5675	0.92(0.86 to 0.99)	
**Drinking Status**				
Never	2983	3398	0.94(0.85 to 1.03)	0.47
Ever	12866	26338	0.90(0.86 to 0.94)	
**Geographic Region**				
Europe	7808	30967	0.91(0.87 to 0.95)	0.6
North America	7555	4829	0.92(0.87 to 0.98)	
South America	3030	2324	0.93(0.85 to 1.02)	
Middle East	505	504	1.00(0.78 to 1.27)	
South Asia	175	233	0.69(0.47 to 1.03)	
**Subsite**				
Oral cavity	5596	37722	0.96(0.92 to 1.01)	0.22
Larynx	4409	37722	0.90(0.85 to 0.95)	
Hypopharynx	898	37722	0.88(0.78 to 0.98)	
Oropharynx (HPV-)	1473	23132	0.88(0.80 to 0.96)	
Oropharynx (HPV+)	2212	22803	0.91(0.85 to 0.98)	

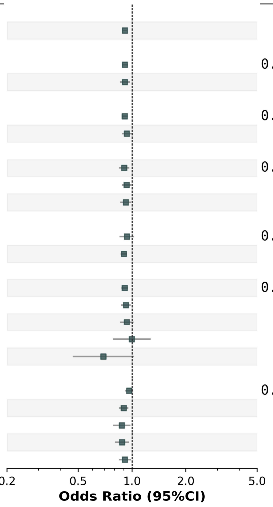

rs78378222

Variable	NO.Case	NO.Control	OR.(95% Conf. Int.)	P-Heterogeneity
**Cross-ancestry meta-analysis**				
All sites combined	19073	38857	0.64(0.54 to 0.75)	
**Genetic Ancestry**				
European	13554	32914	0.62(0.52 to 0.73)	0.3
Mixed*	5519	5943	0.82(0.49 to 1.37)	
**Sex**				
Men	14848	20074	0.64(0.53 to 0.77)	0.87
Women	4225	18783	0.66(0.48 to 0.89)	
**Smoking Status**				
Never	3827	15197	0.61(0.43 to 0.85)	0.15
Former	5811	9741	0.82(0.61 to 1.09)	
Current	6944	5675	0.53(0.36 to 0.77)	
**Drinking Status**				
Never	2983	3398	0.73(0.43 to 1.23)	0.64
Ever	12866	26338	0.64(0.52 to 0.79)	
**Geographic Region**				
Europe	7808	30967	0.56(0.45 to 0.68)	0.25
North America	7555	4829	0.74(0.57 to 0.97)	
South America	3030	2324	0.68(0.34 to 1.38)	
**Subsite**				
Oral cavity	5596	37722	0.51(0.38 to 0.68)	0.02*
Larynx	4409	37722	0.57(0.41 to 0.79)	
Hypopharynx	898	37722	0.69(0.36 to 1.29)	
Oropharynx (HPV-)	1473	23132	0.58(0.35 to 0.96)	
Oropharynx (HPV+)	2212	22803	1.00(0.75 to 1.35)	

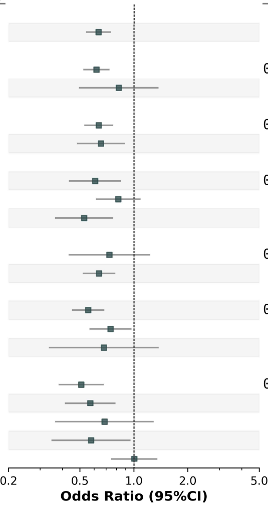

b Cross-ancestry (oral cavity)

rs3846449

Variable	NO.Case	NO.Control	OR.(95% Conf. Int.)	P-Heterogeneity
**Cross-ancestry meta-analysis**				
Oral cavity	5596	37722	0.86(0.82 to 0.91)	
**Genetic ancestry**				
European	3511	31833	0.86(0.81 to 0.92)	0.98
Mixed	2085	5889	0.86(0.79 to 0.94)	
**Sex**				
Men	3749	19246	0.85(0.80 to 0.90)	0.38
women	1847	18476	0.89(0.81 to 0.97)	
**Smoking Status**				
Never	1498	15197	0.94(0.86 to 1.04)	0.05
Former	1598	9741	0.85(0.77 to 0.93)	
Current	2339	5675	0.80(0.73 to 0.88)	
**Drinking Status**				
Never	1218	3398	0.91(0.81 to 1.02)	0.44
Ever	4098	26338	0.87(0.81 to 0.92)	
**Geographic Region**				
Europe	2337	30967	0.89(0.83 to 0.96)	0.1
North America	1770	3694	0.82(0.74 to 0.90)	
South America	1194	2324	0.79(0.71 to 0.89)	
Middle East	153	504	1.09(0.82 to 1.45)	
South Asia	142	233	1.01(0.74 to 1.38)	
**Subsite**				
Larynx	4409	37722	0.94(0.88 to 1.00)	0.0007***
Hypopharynx	898	37722	0.87(0.78 to 0.98)	
Oropharynx (HPV-)	1473	23132	0.77(0.70 to 0.85)	
Oropharynx (HPV+)	2212	22803	0.98(0.91 to 1.06)	

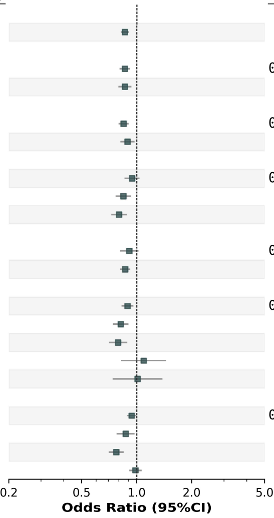

rs1229984

Variable	NO.Case	NO.Control	OR.(95% Conf. Int.)	P-Heterogeneity
**Cross-ancestry meta-analysis**				
Oral cavity	5596	37722	0.74(0.66 to 0.82)	
**Genetic ancestry**				
European	3511	31833	0.65(0.54 to 0.78)	0.1
Mixed	2085	5889	0.79(0.69 to 0.90)	
**Sex**				
Men	3749	19246	0.65(0.56 to 0.74)	0.001**
Women	1847	18476	0.94(0.78 to 1.12)	
**Smoking Status**				
Never	1498	15197	1.08(0.91 to 1.29)	< 0.0001***
Former	1598	9741	0.90(0.74 to 1.10)	
Current	2339	5675	0.43(0.34 to 0.55)	
**Drinking Status**				
Never	1218	3398	1.22(1.01 to 1.46)	< 0.0001***
Ever	4098	26338	0.58(0.50 to 0.68)	
**Geographic Region**				
Europe	2337	30967	0.61(0.50 to 0.73)	0.04*
North America	1770	3694	0.70(0.56 to 0.88)	
South America	1194	2324	0.71(0.56 to 0.90)	
Middle East	153	504	0.98(0.71 to 1.35)	
South Asia	142	233	2.39(0.65 to 8.77)	
**Subsite**				
Larynx	4409	37722	0.80(0.71 to 0.90)	0.001**
Hypopharynx	898	37722	0.57(0.43 to 0.77)	
Oropharynx (HPV-)	948	18166	0.35(0.22 to 0.54)	
Oropharynx (HPV+)	1980	18166	0.82(0.65 to 1.04)	

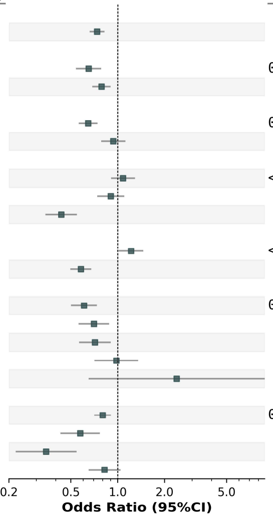

rs7726159

Variable	NO.Case	NO.Control	OR.(95% Conf. Int.)	P-Heterogeneity
**Cross-ancestry meta-analysis**				
Oral cavity	5596	37722	1.18(1.12 to 1.23)	
**Genetic ancestry**				
European	3511	31833	1.18(1.12 to 1.25)	0.86
Mixed	142	233	1.10(0.81 to 1.48)	
**Sex**				
Men	3749	19246	1.15(1.09 to 1.22)	0.17
Women	1847	18476	1.23(1.14 to 1.33)	
**Smoking Status**				
Never	1498	15197	1.25(1.15 to 1.36)	0.28
Former	1598	9741	1.19(1.10 to 1.30)	
Current	2339	5675	1.13(1.04 to 1.23)	
**Drinking Status**				
Never	1218	3398	1.26(1.14 to 1.40)	0.3
Ever	4098	26338	1.19(1.12 to 1.25)	
**Geographic Region**				
Europe	2337	30967	1.19(1.12 to 1.27)	0.99
North America	1770	3694	1.19(1.09 to 1.30)	
South America	1194	2324	1.19(1.08 to 1.33)	
Middle East	153	504	1.15(0.88 to 1.51)	
South Asia	142	233	1.10(0.81 to 1.49)	
**Subsite**				
Larynx	4409	37722	0.98(0.93 to 1.04)	0.46
Hypopharynx	898	37722	1.03(0.93 to 1.14)	
Oropharynx (HPV-)	1473	23132	1.06(0.98 to 1.15)	
Oropharynx (HPV+)	2212	22803	1.01(0.94 to 1.09)	

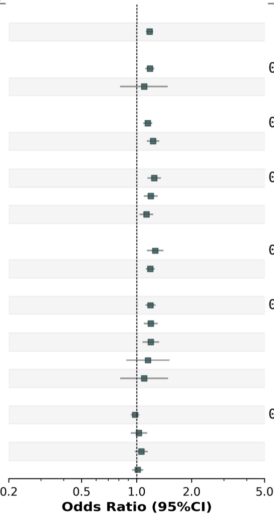

rs60622800

Variable	NO.Case	NO.Control	OR.(95% Conf. Int.)	P-Heterogeneity
**Cross-ancestry meta-analysis**				
Oral cavity	5596	37722	1.19(1.13 to 1.24)	
**Genetic ancestry**				
European	3511	31833	1.20(1.13 to 1.26)	0.65
Mixed	1470	4128	1.17(1.08 to 1.26)	
**Sex**				
Men	3749	19246	1.15(1.09 to 1.21)	0.06
Women	1847	18476	1.25(1.16 to 1.36)	
**Smoking Status**				
Never	1498	15197	1.23(1.13 to 1.34)	0.009**
Former	1598	9741	1.28(1.17 to 1.38)	
Current	2339	5675	1.07(0.98 to 1.16)	
**Drinking Status**				
Never	1218	3398	1.28(1.16 to 1.43)	0.12
Ever	4098	26338	1.17(1.11 to 1.24)	
**Geographic Region**				
Europe	2337	30967	1.14(1.07 to 1.22)	0.05
North America	1770	3694	1.28(1.18 to 1.40)	
South America	1194	2324	1.11(1.00 to 1.23)	
Middle East	153	504	1.37(1.03 to 1.82)	
South Asia	142	233	0.87(0.58 to 1.31)	
**Subsite**				
Larynx	4409	37722	0.97(0.92 to 1.02)	0.5
Hypopharynx	898	37722	0.97(0.87 to 1.07)	
Oropharynx (HPV-)	1473	23132	0.93(0.86 to 1.01)	
Oropharynx (HPV+)	2212	22803	0.91(0.85 to 0.97)	

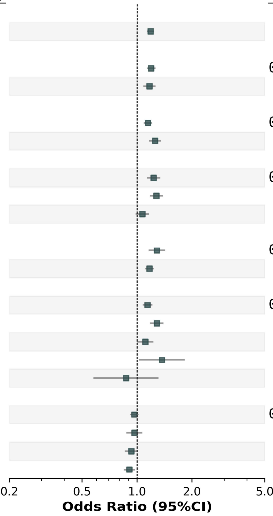

b **Cross-ancestry (oral cavity)**

rs31493

Variable	NO.Case	NO.Control	OR.(95% Conf. Int.)	P-Heterogeneity
**Cross-ancestry meta-analysis**				
Oral cavity	5596	37722	0.85(0.81 to 0.90)	
**Genetic ancestry**				
European	3511	31833	0.85(0.80 to 0.90)	0.85
Mixed	2085	5889	0.86(0.79 to 0.93)	
**Sex**				
Men	3749	19246	0.85(0.80 to 0.90)	0.61
Women	1847	18476	0.87(0.80 to 0.94)	
**Smoking Status**				
Never	1498	15197	0.88(0.80 to 0.96)	0.66
Former	1598	9741	0.83(0.76 to 0.91)	
Current	2339	5675	0.85(0.78 to 0.93)	
**Drinking Status**				
Never	1218	3398	0.81(0.73 to 0.90)	0.29
Ever	4098	26338	0.87(0.82 to 0.92)	
**Geographic Region**				
Europe	2337	30967	0.86(0.80 to 0.92)	0.93
North America	1770	3694	0.89(0.81 to 0.97)	
South America	1194	2324	0.84(0.75 to 0.94)	
Middle East	153	504	0.93(0.70 to 1.24)	
South Asia	142	233	0.90(0.66 to 1.21)	
**Subsite**				
Larynx	4409	37722	0.99(0.94 to 1.05)	0.17
Hypopharynx	898	37722	1.10(0.99 to 1.22)	
Oropharynx (HPV-)	1473	23132	0.95(0.87 to 1.03)	
Oropharynx (HPV+)	2212	22803	1.01(0.94 to 1.09)	

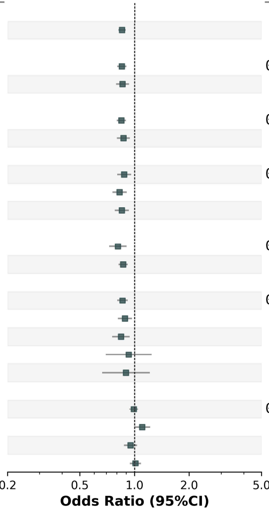

rs28419191

Variable	NO.Case	NO.Control	OR.(95% Conf. Int.)	P-Heterogeneity
**Cross-ancestry meta-analysis**				
Oral cavity	5596	37722	1.23(1.15 to 1.31)	
**Genetic ancestry**				
European	3511	31833	1.22(1.12 to 1.32)	0.77
Mixed	2085	5889	1.24(1.12 to 1.38)	
**Sex**				
Men	3749	19246	1.26(1.17 to 1.36)	0.18
Women	1847	18476	1.15(1.03 to 1.29)	
**Smoking Status**				
Never	1498	15197	1.16(1.03 to 1.31)	
Former	1598	9741	1.24(1.10 to 1.39)	0.74
Current	2339	5675	1.23(1.09 to 1.39)	
**Drinking Status**				
Never	1218	3398	1.23(1.06 to 1.43)	0.99
Ever	4098	26338	1.23(1.14 to 1.33)	
**Geographic Region**				
Europe	2337	30967	1.18(1.08 to 1.29)	0.79
North America	1770	3694	1.26(1.12 to 1.42)	
South America	1194	2324	1.28(1.11 to 1.48)	
Middle East	153	504	1.30(0.89 to 1.92)	
South Asia	142	233	1.04(0.60 to 1.80)	
**Subsite**				
Larynx	4409	37722	1.15(1.07 to 1.24)	0.01*
Hypopharynx	898	37722	1.12(0.97 to 1.29)	
Oropharynx (HPV-)	1473	23132	1.14(1.02 to 1.28)	
Oropharynx (HPV+)	2212	22803	0.94(0.85 to 1.04)	

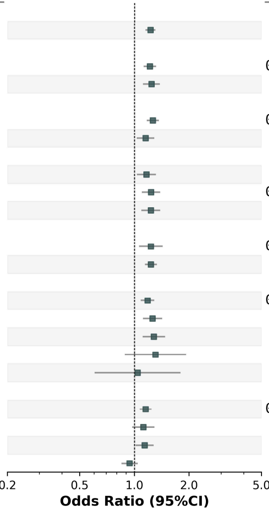

rs9271300

Variable	NO.Case	NO.Control	OR.(95% Conf. Int.)	P-Heterogeneity
**Cross-ancestry meta-analysis**				
Oral cavity	5596	37722	0.84(0.80 to 0.88)	
**Genetic ancestrt**				
European	3511	31833	0.83(0.78 to 0.87)	0.32
Mixed	2085	5889	0.87(0.81 to 0.93)	
**Sex**				
Men	3749	19246	0.82(0.78 to 0.86)	0.09
Women	1847	18476	0.89(0.82 to 0.96)	
**Smoking Status**				
Never	1498	15197	0.85(0.78 to 0.92)	0.2
Former	1598	9741	0.80(0.74 to 0.87)	
Current	2339	5675	0.89(0.82 to 0.96)	
**Drinking Status**				
Never	1218	3398	0.81(0.73 to 0.89)	0.26
Ever	4098	26338	0.86(0.82 to 0.91)	
**Geographic Region**				
Europe	2337	30967	0.85(0.80 to 0.90)	0.78
North America	1770	3694	0.84(0.77 to 0.91)	
South America	1194	2324	0.89(0.80 to 0.98)	
Middle East	153	504	0.95(0.72 to 1.24)	
South Asia	142	233	0.93(0.69 to 1.24)	
**Subsite**				
Larynx	4409	37722	0.95(0.91 to 1.00)	0.2
Hypopharynx	898	37722	0.92(0.83 to 1.01)	
Oropharynx (HPV-)	1473	23132	0.87(0.80 to 0.94)	
Oropharynx (HPV+)	2212	22803	0.90(0.84 to 0.96)	

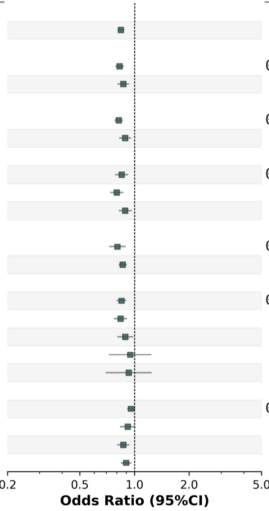

rs407238

Variable	NO.Case	NO.Control	OR.(95% Conf. Int.)	P-Heterogeneity
**Cross-ancestry meta-analysis**				
Oral cavity	5596	37722	1.15(1.10 to 1.20)	
**Genetic ancestry**				
European	3511	31833	1.20(1.14 to 1.27)	0.004**
Mixed	2085	5889	1.04(0.97 to 1.13)	
**Sex**				
Men	3749	19246	1.14(1.08 to 1.20)	0.51
Women	1847	18476	1.17(1.09 to 1.27)	
**Smoking Status**				
Never	1498	15197	1.14(1.05 to 1.24)	0.33
Former	1598	9741	1.19(1.10 to 1.29)	
Current	2339	5675	1.09(1.00 to 1.18)	
**Drinking Status**				
Never	1218	3398	1.14(1.03 to 1.27)	0.79
Ever	4098	26338	1.16(1.10 to 1.22)	
**Cluster**				
AFR	125	273	1.15(0.81 to 1.62)	0.01*
SAS	184	488	0.96(0.74 to 1.24)	
EAS	28	24	0.76(0.27 to 2.15)	
EUR	3511	31833	1.20(1.14 to 1.27)	
AMR	6	29	3.33(0.60 to 18.43)	
Admixed	1742	5075	1.02(0.94 to 1.10)	
**Geographic Region**				
Europe	2337	30967	1.18(1.11 to 1.25)	0.37
North America	1770	3694	1.13(1.04 to 1.23)	
South America	1194	2324	1.07(0.96 to 1.19)	
Middle East	153	504	0.96(0.73 to 1.26)	
South Asia	142	233	1.12(0.81 to 1.54)	
**Subsite**				
Larynx	4409	37722	1.08(1.02 to 1.14)	0.01*
Hypopharynx	332	5889	0.92(0.78 to 1.10)	
Oropharynx (HPV-)	1473	23132	1.00(0.92 to 1.08)	
Oropharynx (HPV+)	2212	22803	0.95(0.89 to 1.02)	

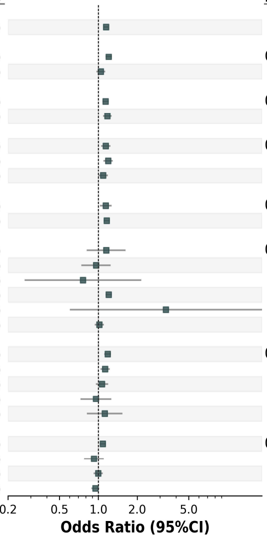

b **Cross-ancestry (oral cavity)**

rs3731239

Variable	NO.Case	NO.Control	OR.(95% Conf. Int.)	P-Heterogeneity
**Cross-ancestry meta-analysis**				
Oral cavity	5596	37722	1.14(1.09 to 1.19)	
**Genetic ancestry**				
European	3511	31833	1.14(1.08 to 1.20)	1
Mixed	2085	5889	1.14(1.05 to 1.24)	
**Sex**				
Men	3749	19246	1.16(1.09 to 1.22)	0.39
Women	1847	18476	1.11(1.02 to 1.20)	
**Smoking Status**				
Never	1498	15197	1.12(1.03 to 1.22)	0.65
Former	1598	9741	1.16(1.07 to 1.27)	
Current	2339	5675	1.19(1.08 to 1.30)	
**Drinking Status**				
Never	1218	3398	1.14(1.02 to 1.27)	0.91
Ever	4098	26338	1.15(1.09 to 1.22)	
**Geographic Region**				
Europe	2337	30967	1.14(1.07 to 1.21)	0.14
North America	1770	3694	1.17(1.07 to 1.27)	
South America	1194	2324	1.21(1.08 to 1.36)	
Middle East	153	504	0.88(0.65 to 1.19)	
South Asia	142	233	0.83(0.57 to 1.21)	
**Subsite**				
Larynx	4409	37722	1.09(1.04 to 1.16)	0.005**
Hypopharynx	898	37722	0.93(0.83 to 1.03)	
Oropharynx (HPV-)	1473	23132	1.08(1.00 to 1.18)	
Oropharynx (HPV+)	2212	22803	0.97(0.91 to 1.04)	

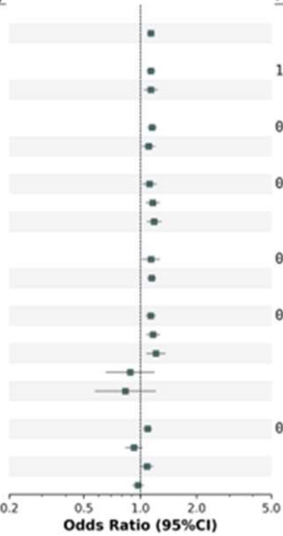

rs12910284

Variable	NO.Case	NO.Control	OR.(95% Conf. Int.)	P-Heterogeneity
**Cross-ancestry meta-analysis**				
Oral cavity	5596	37722	1.17(1.12 to 1.23)	
**Genetic ancestry**				
European	3511	31833	1.20(1.14 to 1.27)	0.13
Mixed	2085	5889	1.11(1.03 to 1.21)	
**Sex**				
Men	3749	19246	1.19(1.13 to 1.26)	0.25
Women	1847	18476	1.13(1.04 to 1.22)	
**Smoking Status**				
Never	1498	15197	1.20(1.10 to 1.30)	0.7
Former	1598	9741	1.14(1.05 to 1.24)	
Current	2339	5675	1.19(1.09 to 1.29)	
**Drinking Status**				
Never	1218	3398	1.12(1.00 to 1.24)	0.42
Ever	4098	26338	1.17(1.11 to 1.24)	
**Geographic Region**				
Europe	2337	30967	1.15(1.08 to 1.22)	0.92
North America	1770	3694	1.18(1.08 to 1.28)	
South America	1194	2324	1.11(0.99 to 1.24)	
Middle East	153	504	1.07(0.79 to 1.44)	
South Asia	142	233	1.13(0.80 to 1.59)	
**Subsite**				
Larynx	4409	37722	1.02(0.97 to 1.07)	0.11
Hypopharynx	898	37722	0.98(0.89 to 1.09)	
Oropharynx (HPV-)	1473	23132	1.12(1.03 to 1.21)	
Oropharynx (HPV+)	2212	22803	1.00(0.93 to 1.07)	

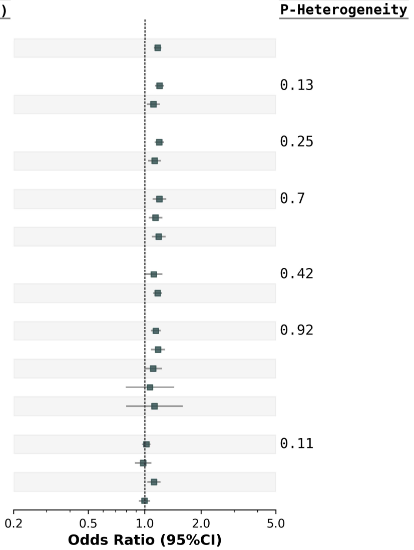

rs67351073

Variable	NO.Case	NO.Control	OR.(95% Conf. Int.)	P-Heterogeneity
**Cross-ancestry meta-analysis**				
Oral cavity	5596	37722	0.78(0.72 to 0.85)	
**Genetic ancestry**				
European	3511	31833	0.75(0.67 to 0.83)	0.13
Mixed	2085	5889	0.86(0.74 to 1.01)	
**Sex**				
Men	3749	19246	0.76(0.69 to 0.85)	0.61
Women	1847	18476	0.80(0.69 to 0.93)	
**Smoking Status**				
Never	1498	15197	0.76(0.64 to 0.90)	0.86
Former	1598	9741	0.79(0.68 to 0.93)	
Current	2339	5675	0.75(0.63 to 0.88)	
**Drinking Status**				
Never	1218	3398	0.91(0.74 to 1.11)	0.15
Ever	4098	26338	0.77(0.69 to 0.85)	
**Geographic Region**				
Europe	2337	30967	0.76(0.67 to 0.86)	
North America	1770	3694	0.76(0.65 to 0.89)	0.71
South America	1194	2324	0.85(0.69 to 1.05)	
Middle East	153	504	1.13(0.54 to 2.37)	
South Asia	142	233	0.63(0.31 to 1.28)	
**Subsite**				
Larynx	4409	37722	0.99(0.90 to 1.10)	0.42
Hypopharynx	898	37722	0.91(0.75 to 1.10)	
Oropharynx (HPV-)	1473	23132	0.94(0.81 to 1.09)	
Oropharynx (HPV+)	2212	22803	1.07(0.95 to 1.20)	

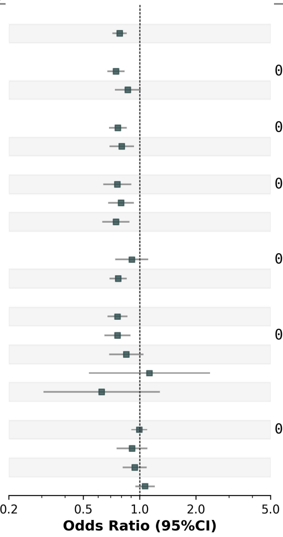

rs577454702

Variable	NO.Case	NO.Control	OR.(95% Conf. Int.)	P-Heterogeneity
**Cross-ancestry meta-analysis**				
Oral cavity	5596	37722	2.51(1.86 to 3.39)	
**Genetic ancestry**				
European	3511	31833	2.60(1.86 to 3.65)	0.64
Mixed	2085	5889	2.18(1.13 to 4.22)	
**Sex**				
Men	3749	19246	2.29(1.56 to 3.37)	0.49
Women	1847	18476	2.84(1.75 to 4.61)	
**Smoking Status**				
Never	1498	15197	3.31(2.00 to 5.46)	0.51
Former	1598	9741	2.15(1.23 to 3.77)	
Current	2339	5675	2.46(1.29 to 4.69)	
**Drinking Status**				
Never	1218	3398	2.90(1.31 to 6.43)	0.96
Ever	4098	26338	2.96(2.06 to 4.27)	
**Geographic Region**				
Europe	2337	30967	2.24(1.56 to 3.22)	0.94
North America	1770	3694	2.02(1.11 to 3.69)	
South America	1123	2239	2.36(1.04 to 5.33)	
**Subsite**				
Larynx	4409	37722	1.04(0.69 to 1.58)	0.55
Hypopharynx	898	37722	1.48(0.65 to 3.33)	
Oropharynx (HPV-)	1473	23132	1.32(0.67 to 2.60)	
Oropharynx (HPV+)	1980	18166	1.68(0.99 to 2.84)	

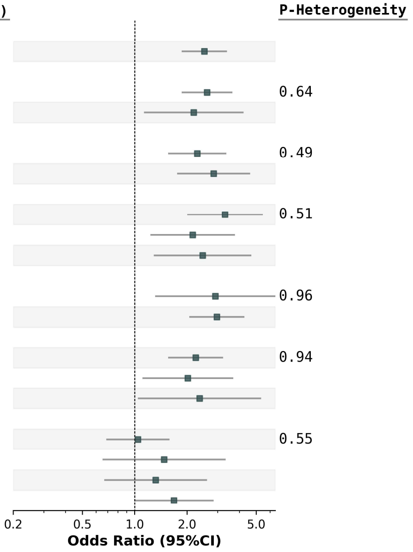

c Cross-ancestry (larynx)

rs10774632

Variable	NO.Case	NO.Control	OR.(95% Conf. Int.)	P-Heterogeneity
**Cross-ancestry meta-analysis**				
Larynx	4409	37722	1.20(1.13 to 1.28)	
**Genetic ancestry**				
European	2787	31833	1.24(1.15 to 1.34)	0.2
Mixed	1622	5889	1.14(1.03 to 1.26)	
**Sex**				
Men	3826	19246	1.17(1.09 to 1.25)	0.12
Women	583	18476	1.33(1.15 to 1.55)	
**Smoking Status**				
Never	552	15197	1.09(0.90 to 1.32)	0.55
Former	1726	9741	1.20(1.07 to 1.33)	
Current	2114	5675	1.24(1.11 to 1.38)	
**Drinking Status**				
Never	774	3398	1.17(1.00 to 1.37)	0.86
Ever	3366	26338	1.19(1.10 to 1.28)	
**Geographic Region**				
Europe	2003	30967	1.18(1.08 to 1.28)	0.16
North America	1354	3694	1.22(1.09 to 1.37)	
South America	770	2324	1.01(0.89 to 1.15)	
Middle East	282	504	1.11(0.86 to 1.44)	
**Subsite**				
Oral cavity	5596	37722	1.04(0.98 to 1.09)	0.0003***
Hypopharynx	898	37722	1.20(1.07 to 1.34)	
Oropharynx (HPV-)	1473	23132	1.23(1.12 to 1.34)	
Oropharynx (HPV+)	2212	22803	0.96(0.88 to 1.05)	

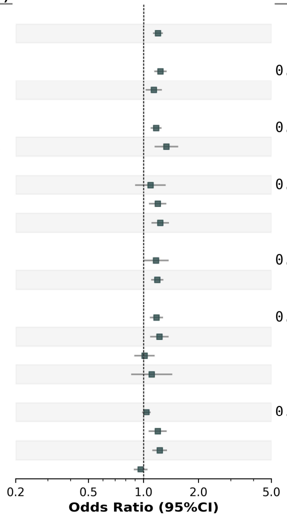

rs11571833

Variable	NO.Case	NO.Control	OR.(95% Conf. Int.)	P-Heterogeneity
**Cross-ancestry meta-analysis**				
Larynx	4409	37722	2.09(1.65 to 2.66)	
**Genetic ancestry**				
European	2787	31833	2.12(1.62 to 2.78)	0.81
Mixed	1622	5889	1.98(1.17 to 3.34)	
**Sex**				
Men	3826	19246	2.16(1.66 to 2.81)	0.69
Women	583	18476	1.89(1.02 to 3.51)	
**Smoking Status**				
Never	552	15197	3.02(1.73 to 5.28)	0.35
Former	1726	9741	1.89(1.18 to 3.04)	
Current	2114	5675	2.91(1.74 to 4.86)	
**Drinking Status**				
Never	774	3398	2.06(1.08 to 3.95)	0.73
Ever	3366	26338	2.34(1.70 to 3.22)	
**Geographic Region**				
Europe	2003	30967	2.00(1.51 to 2.64)	
North America	1354	3694	2.20(1.42 to 3.41)	0.87
South America	770	2324	1.75(0.87 to 3.49)	
Middle East	282	504	1.37(0.42 to 4.48)	
**Subsite**				
Oral cavity	5596	37722	1.63(1.32 to 2.02)	0.009**
Hypopharynx	898	37722	2.73(1.91 to 3.90)	
Oropharynx (HPV-)	1473	23132	2.48(1.82 to 3.38)	
Oropharynx (HPV+)	2212	22803	1.45(1.06 to 1.97)	

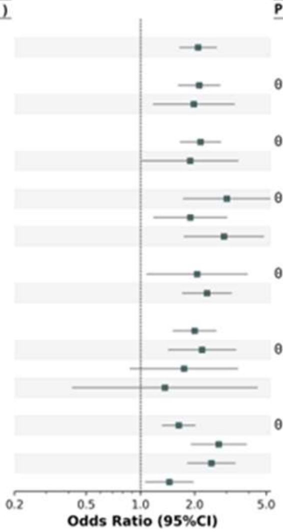

rs10419397

Variable	NO.Case	NO.Control	OR.(95% Conf. Int.)	P-Heterogeneity
**Cross-ancestry meta-analysis**				
Larynx	4409	37722	1.17(1.11 to 1.24)	
**Genetic ancestry**				
European	2787	31833	1.18(1.10 to 1.26)	0.66
Mixed	1622	5889	1.15(1.03 to 1.27)	
**Sex**				
Men	3826	19246	1.16(1.09 to 1.24)	0.57
Women	583	18476	1.22(1.06 to 1.40)	
**Smoking Status**				
Never	552	15197	1.19(1.00 to 1.43)	0.8
Former	1726	9741	1.14(1.03 to 1.26)	
Current	2114	5675	1.19(1.08 to 1.31)	
**Drinking Status**				
Never	774	3398	1.13(0.96 to 1.32)	0.81
Ever	3366	26338	1.15(1.07 to 1.24)	
**Geographic Region**				
Europe	2003	30967	1.19(1.11 to 1.27)	0.78
North America	1354	3694	1.19(1.08 to 1.32)	
South America	770	2324	1.10(0.96 to 1.26)	
Middle East	282	504	1.17(0.90 to 1.52)	
**Subsite**				
Oral cavity	5596	37722	1.09(1.04 to 1.14)	0.59
Hypopharynx	898	37722	1.17(1.05 to 1.30)	
Oropharynx (HPV-)	1473	23132	1.07(0.98 to 1.17)	
Oropharynx (HPV+)	2212	22803	1.09(1.01 to 1.17)	

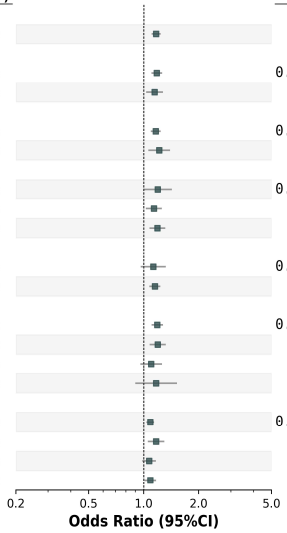

d Cross-ancestry (hypopharynx)

rs1154462

Variable	NO.Case	NO.Control	OR.(95% Conf. Int.)	P-Heterogeneity
**Cross-ancestry meta-analysis**				
Hypopharynx	898	37722	1.34(1.22 to 1.49)	
**Genetic ancestry**				
European	566	31833	1.38(1.22 to 1.56)	0.52
Mixed	332	5889	1.28(1.08 to 1.53)	
**Sex**				
Men	759	19246	1.38(1.24 to 1.55)	0.2
Women	139	18476	1.16(0.90 to 1.49)	
**Smoking Status**				
Never	151	15197	1.40(1.08 to 1.81)	0.05
Former	297	9741	1.10(0.92 to 1.32)	
Current	439	5675	1.47(1.26 to 1.72)	
**Drinking Status**				
Never	99	3398	1.56(1.13 to 2.16)	0.28
Ever	739	26338	1.30(1.16 to 1.46)	
**Geographic Region**				
Europe	426	30967	1.30(1.13 to 1.49)	0.13
North America	238	3694	1.64(1.34 to 2.01)	
South America	188	2324	1.16(0.91 to 1.47)	
Middle East	46	504	1.50(0.95 to 2.37)	
**Subsite**				
Oral cavity	5596	37722	1.07(1.02 to 1.12)	0.41
Larynx	4409	37722	1.05(1.00 to 1.11)	
Oropharynx (HPV-)	1473	23132	1.13(1.04 to 1.23)	
Oropharynx (HPV+)	2212	22803	1.03(0.96 to 1.11)	

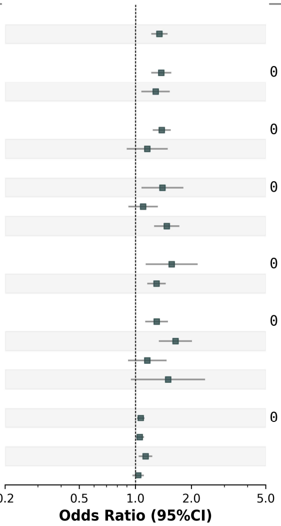

rs11571815

Variable	NO.Case	NO.Control	OR.(95% Conf. Int.)	P-Heterogeneity
**Cross-ancestry meta-analysis**				
Hypopharynx	898	37722	2.73(1.91 to 3.90)	
**Genetic ancestry**				
European	566	31833	2.93(1.97 to 4.37)	0.42
Mixed	332	5889	2.03(0.91 to 4.54)	
**Sex**				
Men	759	19246	2.67(1.80 to 3.97)	0.8
Women	139	18476	3.00(1.29 to 6.98)	
**Smoking Status**				
Never	151	15197	1.80(0.53 to 6.10)	0.58
Former	297	9741	3.65(1.99 to 6.71)	
Current	439	5675	3.54(1.90 to 6.59)	
**Drinking Status**				
Never	99	3398	2.47(0.75 to 8.12)	0.81
Ever	739	26338	2.89(1.92 to 4.35)	
**Geographic Region**				
Europe	426	30967	2.90(1.83 to 4.60)	0.62
North America	179	2937	4.16(2.07 to 8.38)	
South America	184	2239	2.33(0.78 to 6.92)	
Middle East	46	504	1.18(0.13 to 10.32)	
**Subsite**				
Oral cavity	5596	37722	1.63(1.32 to 2.02)	0.04*
Larynx	4409	37722	2.09(1.64 to 2.66)	
Oropharynx (HPV-)	1473	23132	2.48(1.82 to 3.37)	
Oropharynx (HPV+)	2212	22803	1.45(1.06 to 1.97)	

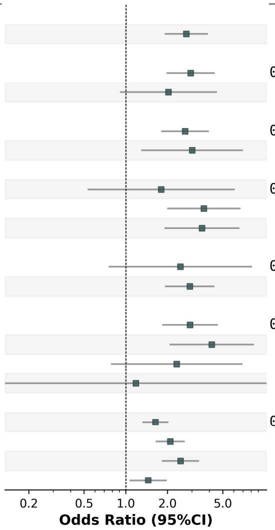

rs181194133

Variable	NO.Case	NO.Control	OR.(95% Conf. Int.)	P-Heterogeneity
**Cross-ancestry meta-analysis**				
Hypopharynx	898	37722	3.24(2.12 to 4.96)	
**Genetic ancestry**				
European	566	31833	3.44(2.23 to 5.31)	0.15
Mixed	332	5889	0.66(0.07 to 6.13)	
**Sex**				
Men	759	19246	2.98(1.79 to 4.99)	0.39
Women	139	18476	4.45(2.09 to 9.46)	
**Smoking Status**				
Never	151	15197	3.15(1.23 to 8.07)	0.96
Former	206	8018	3.75(1.73 to 8.13)	
Current	439	5675	3.58(1.69 to 7.59)	
**Drinking Status**				
Never	99	3398	3.48(0.78 to 15.60)	0.92
Ever	463	22669	3.77(2.30 to 6.17)	
**Geographic Region**				
Europe	383	28811	2.53(1.46 to 4.38)	0.16
North America	179	2937	6.86(3.09 to 15.23)	
Middle East	46	504	2.85(0.41 to 19.75)	
**Subsite**				
Larynx	4409	37722	1.10(0.79 to 1.52)	0.97
Oral cavity	5596	37722	1.22(0.92 to 1.62)	
Oropharynx (HPV-)	1473	23132	1.19(0.72 to 1.99)	
Oropharynx (HPV+)	2212	22803	1.15(0.78 to 1.70)	

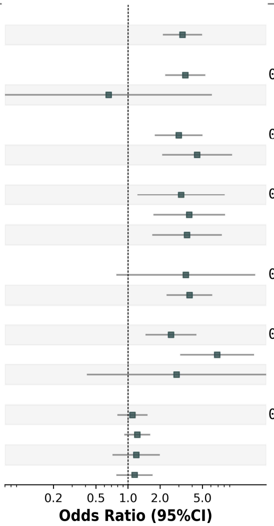

Supplementary Figure 7. Forest plots of odds ratios for independent top hits from the cross-ancestry GWAS, by cancer subsite. Analyses were stratified by sex, smoking status, drinking status, geographic region, and cancer subsites. a) top hit variants identified in all sites combined, b) variants identified in the oral cavity, c) variants identified in the larynx, and d) variants identified in the hypopharynx.

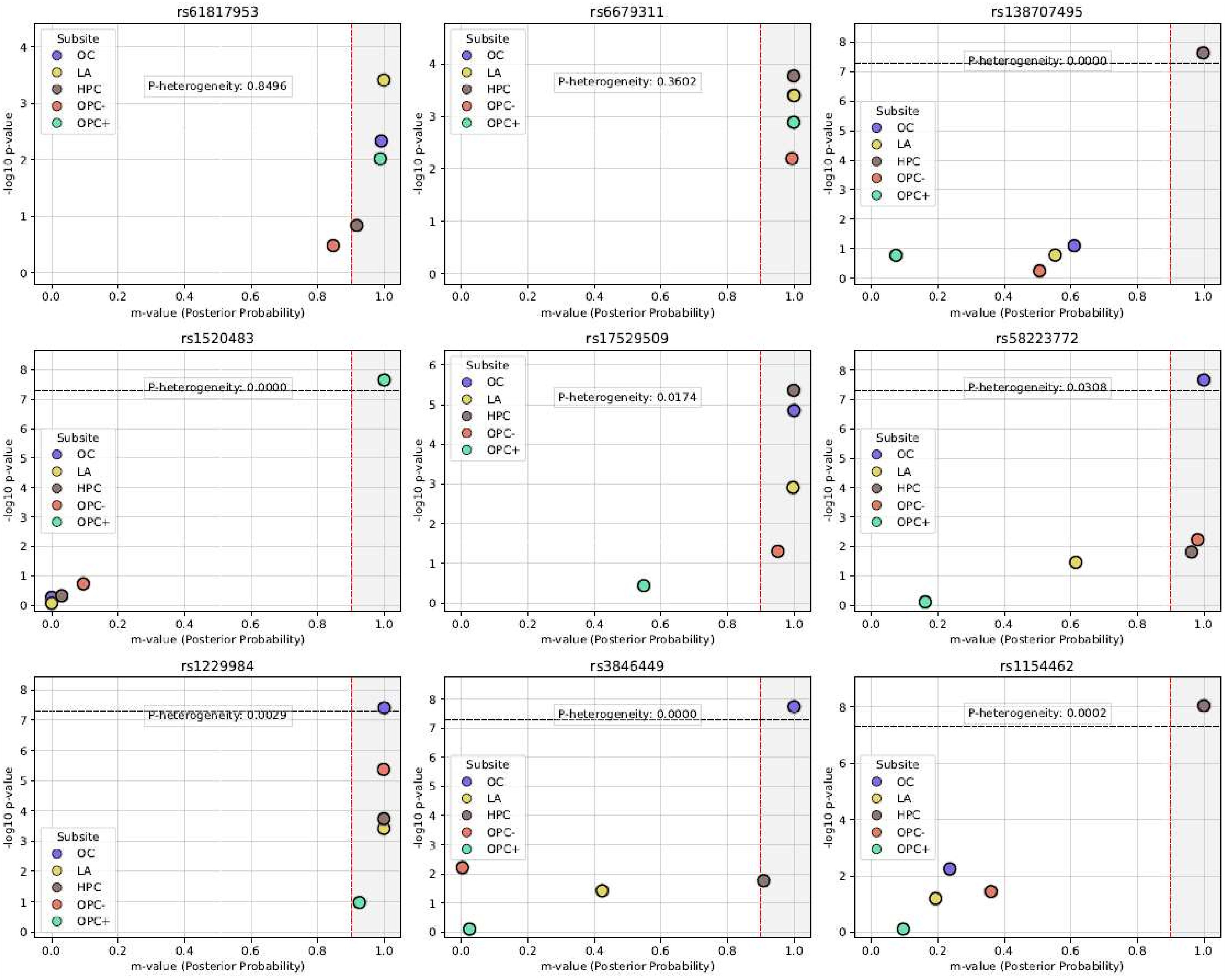
rs61817953
P-heterogeneity: 0.8496
rs6679311
P-heterogeneity: 0.3602
rs138707495
P-heterogeneity: 0.0000
rs1520483
P-heterogeneity: 0.0000
rs17529509
P-heterogeneity: 0.0174
rs58223772
P-heterogeneity: 0.0308
rs1229984
P-heterogeneity: 0.0029
rs3846449
P-heterogeneity: 0.0000
rs1154462
P-heterogeneity: 0.0002
Subsite
OC
LA
HPC
OPC-
OPC+
-log10 p-value
m-value (Posterior Probability)

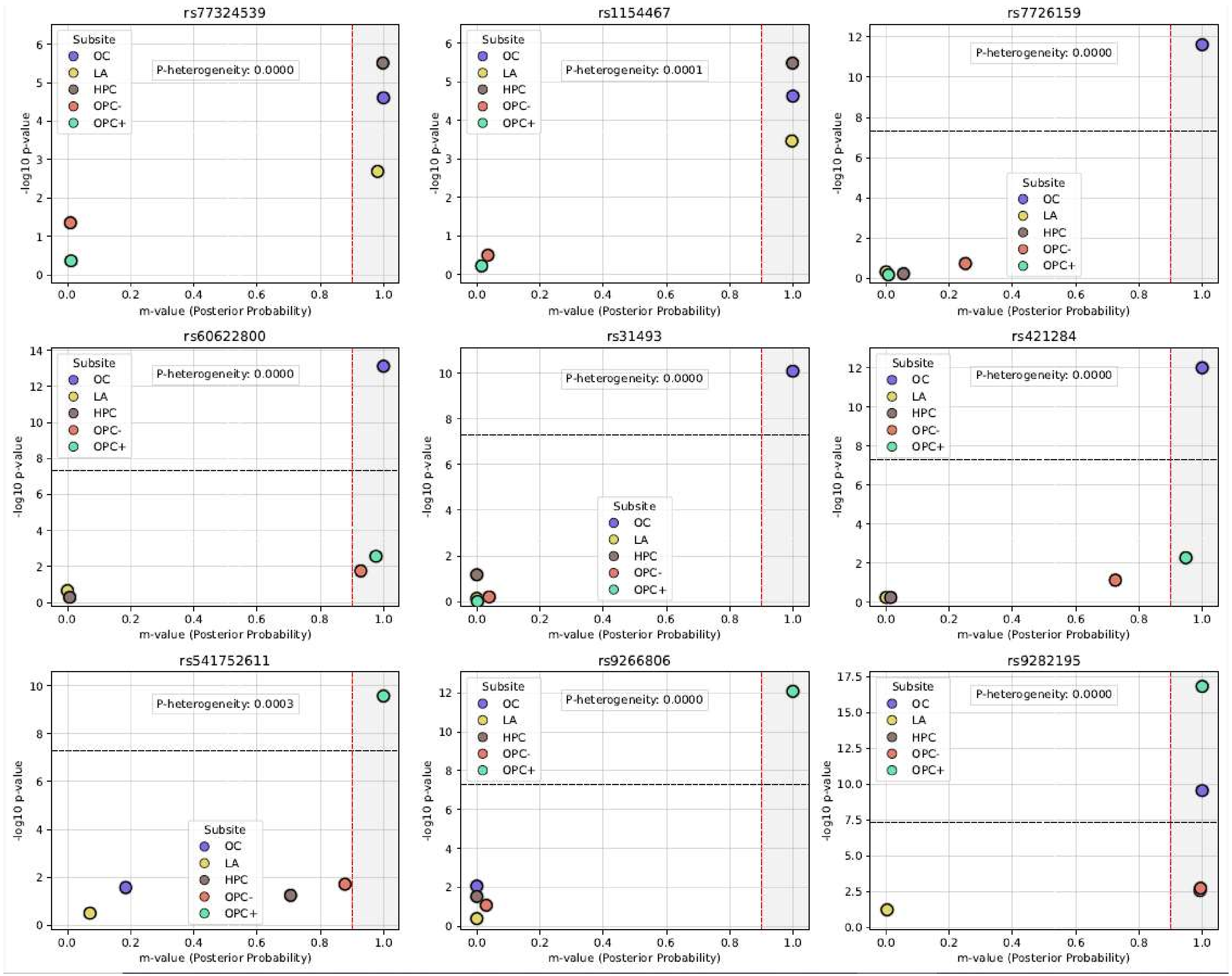
rs77324539
P-heterogeneity: 0.0000
rs1154467
P-heterogeneity: 0.0001
rs7726159
P-heterogeneity: 0.0000
rs60622800
P-heterogeneity: 0.0000
rs31493
P-heterogeneity: 0.0000
rs421284
P-heterogeneity: 0.0000
rs541752611
P-heterogeneity: 0.0003
rs9266806
P-heterogeneity: 0.0000
rs9282195
P-heterogeneity: 0.0000
Subsite
OC
LA
HPC
OPC-
OPC+
-log10 p-value
m-value (Posterior Probability)

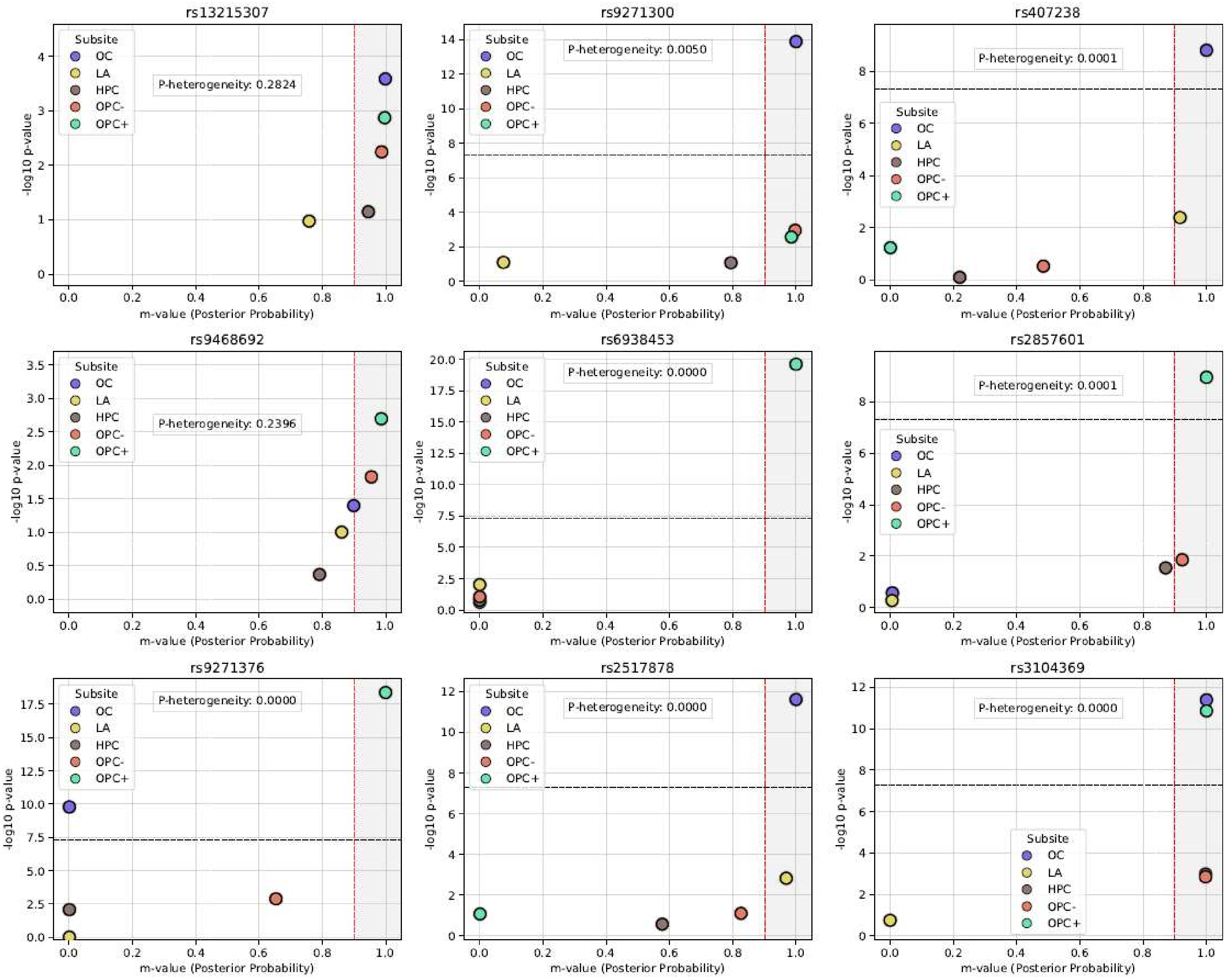
rs13215307
Subsite
OC
LA
HPC
OPC-
OPC+
P-heterogeneity: 0.2824
-log10 p-value
m-value (Posterior Probability)
rs9271300
P-heterogeneity: 0.0050
rs407238
P-heterogeneity: 0.0001
rs9468692
P-heterogeneity: 0.2396
rs6938453
P-heterogeneity: 0.0000
rs2857601
P-heterogeneity: 0.0001
rs9271376
P-heterogeneity: 0.0000
rs2517878
P-heterogeneity: 0.0000
rs3104369
P-heterogeneity: 0.0000

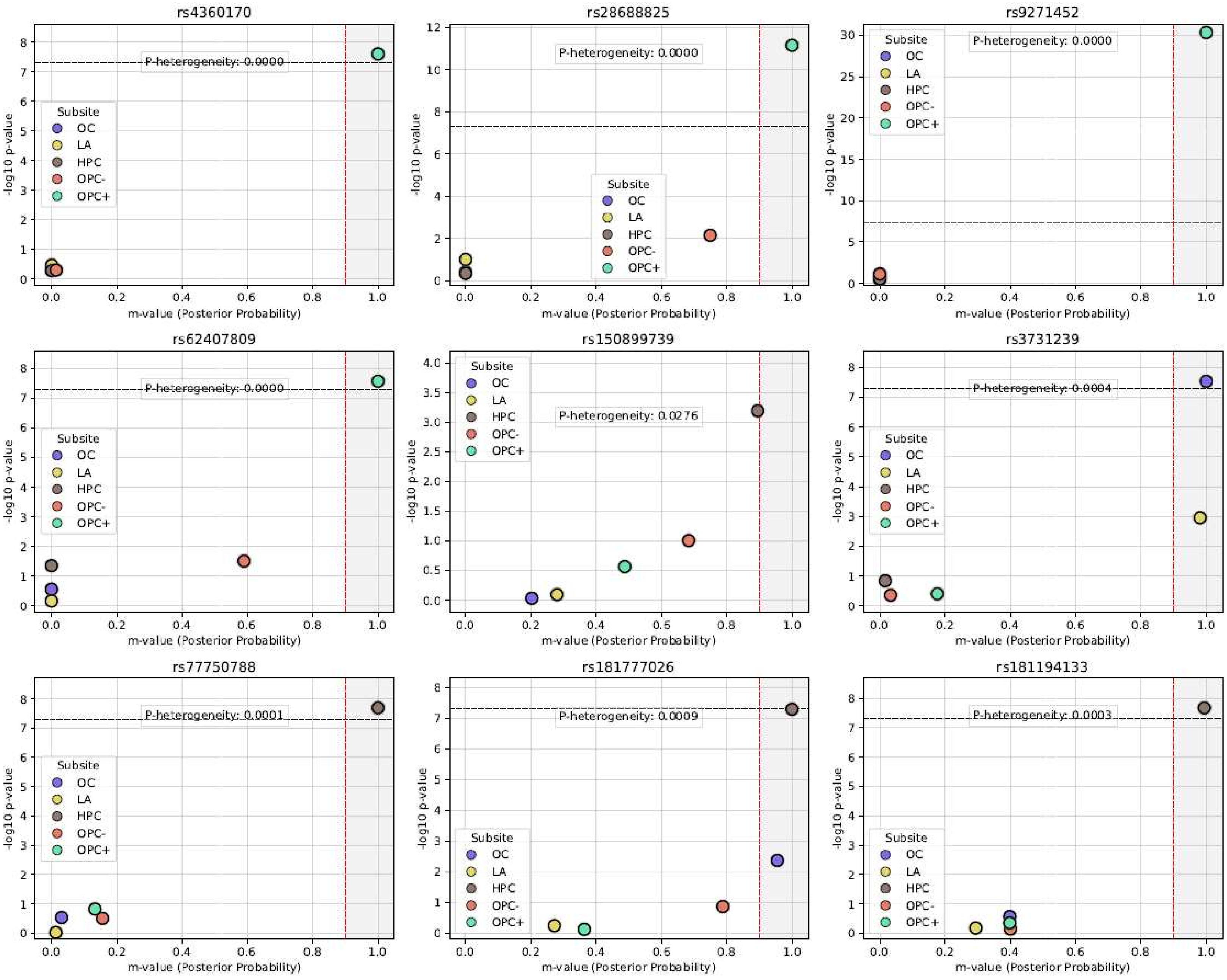
rs4360170
P-heterogeneity: 0.0000
rs28688825
P-heterogeneity: 0.0000
rs9271452
P-heterogeneity: 0.0000
rs62407809
P-heterogeneity: 0.0000
rs150899739
P-heterogeneity: 0.0276
rs3731239
P-heterogeneity: 0.0004
rs77750788
P-heterogeneity: 0.0001
rs181777026
P-heterogeneity: 0.0009
rs181194133
P-heterogeneity: 0.0003
Subsite
OC
LA
HPC
OPC-
OPC+
-log10 p-value
m-value (Posterior Probability)

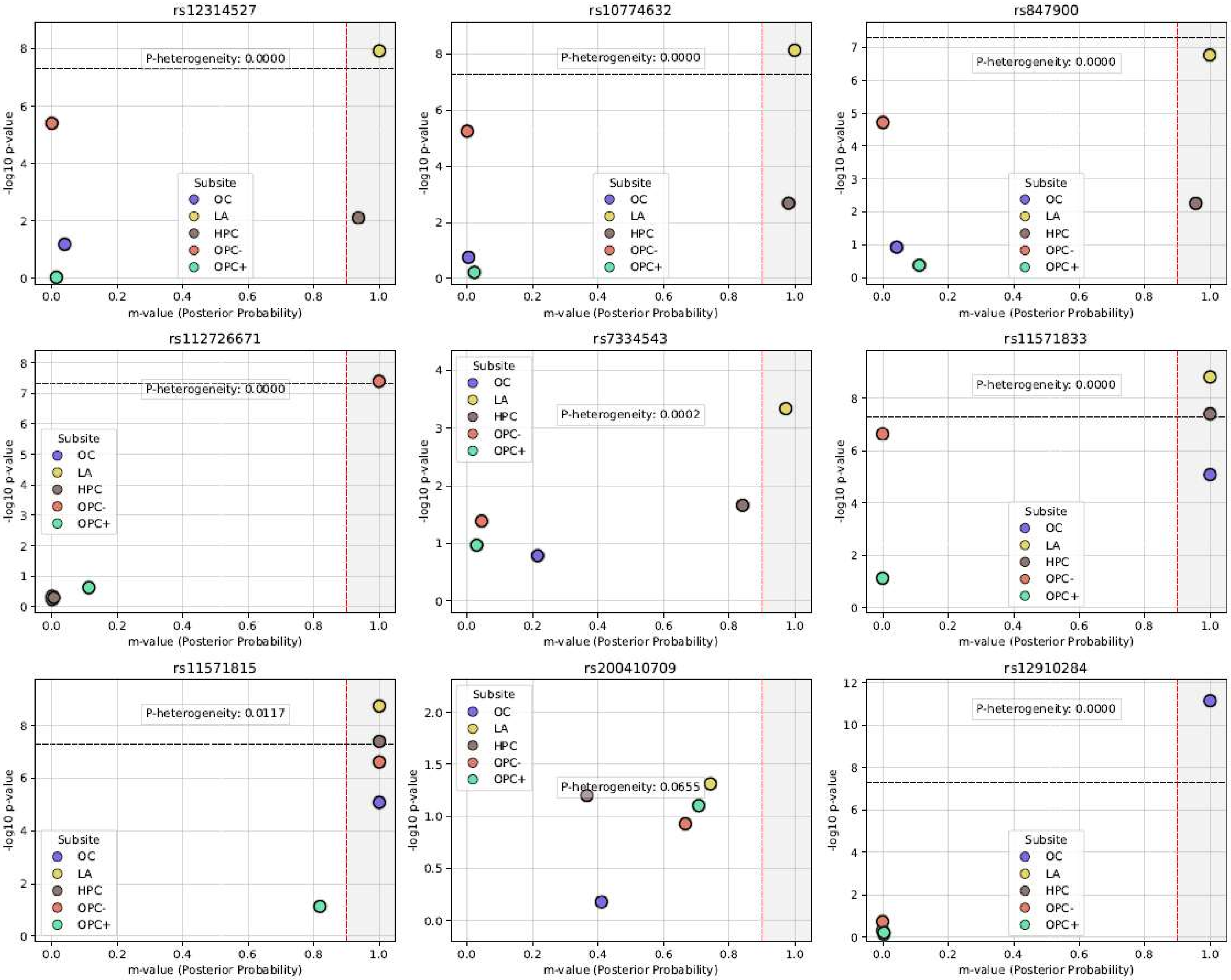
rs12314527
P-heterogeneity: 0.0000
rs10774632
P-heterogeneity: 0.0000
rs847900
P-heterogeneity: 0.0000
rs112726671
P-heterogeneity: 0.0000
rs7334543
P-heterogeneity: 0.0002
rs11571833
P-heterogeneity: 0.0000
rs11571815
P-heterogeneity: 0.0117
rs200410709
P-heterogeneity: 0.0655
rs12910284
P-heterogeneity: 0.0000
Subsite
OC
LA
HPC
OPC-
OPC+
-log10 p-value
m-value (Posterior Probability)

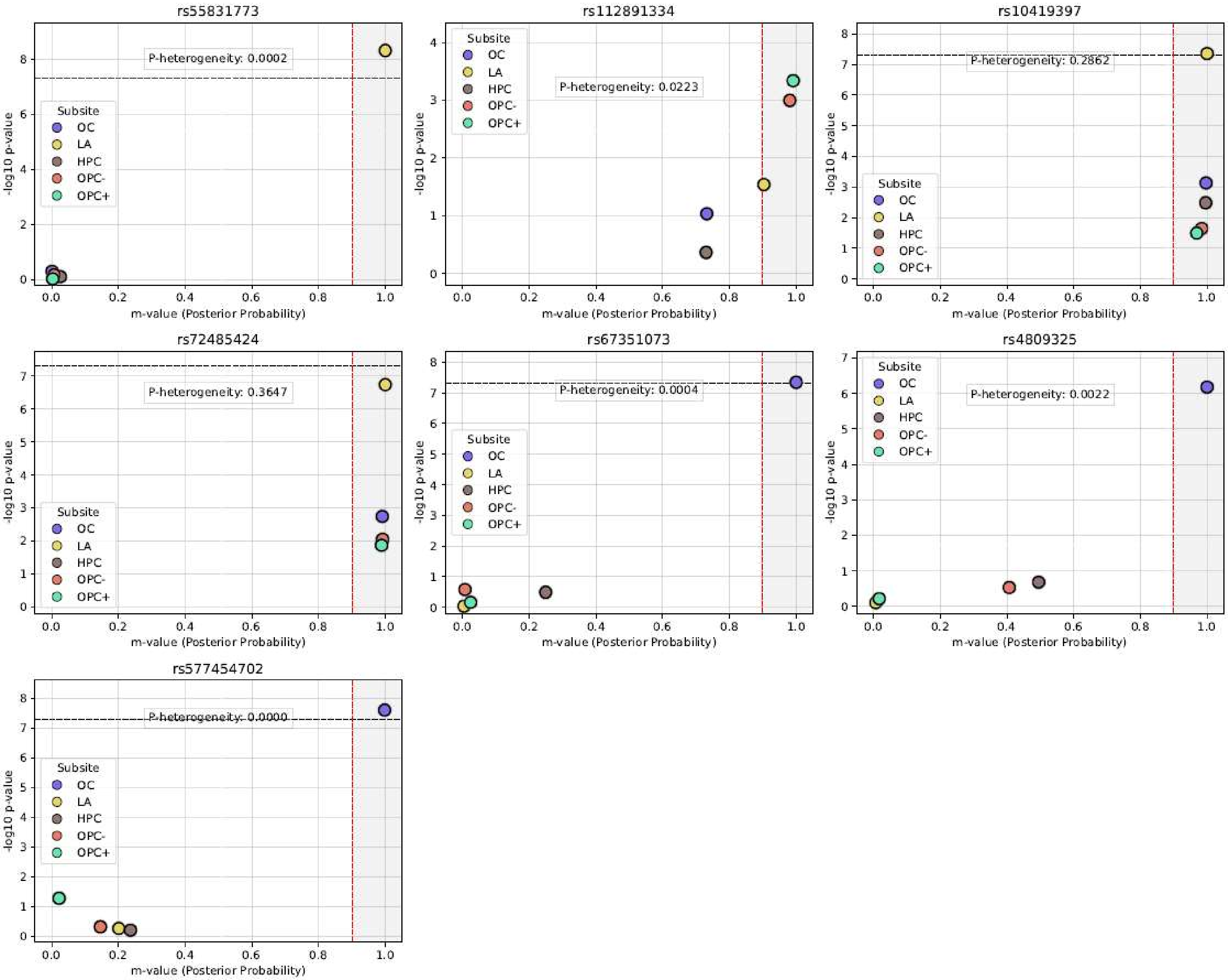

Supplementary Figure 8. PM-plots of identified variants in cross-ancestry meta-analysis using METASOFT. PM-plots display the m-value on the x-axis, representing the posterior probability that a given subsite has a risk effect, and the $-\log_{10}$ p-value on the y-axis, which reflects the statistical significance of the association obtained in the subsite-specific meta-analysis. The m-value ranges from 0 to 1. An m-value below 0.1 indicates that the subsite likely does not have an effect on HNSCC risk, while an m-value above 0.9 indicates that the subsite likely does have an effect. Values between 0.1 and 0.9 signify uncertainty regarding the presence of a risk effect. Source data are provided as a Source Data file.

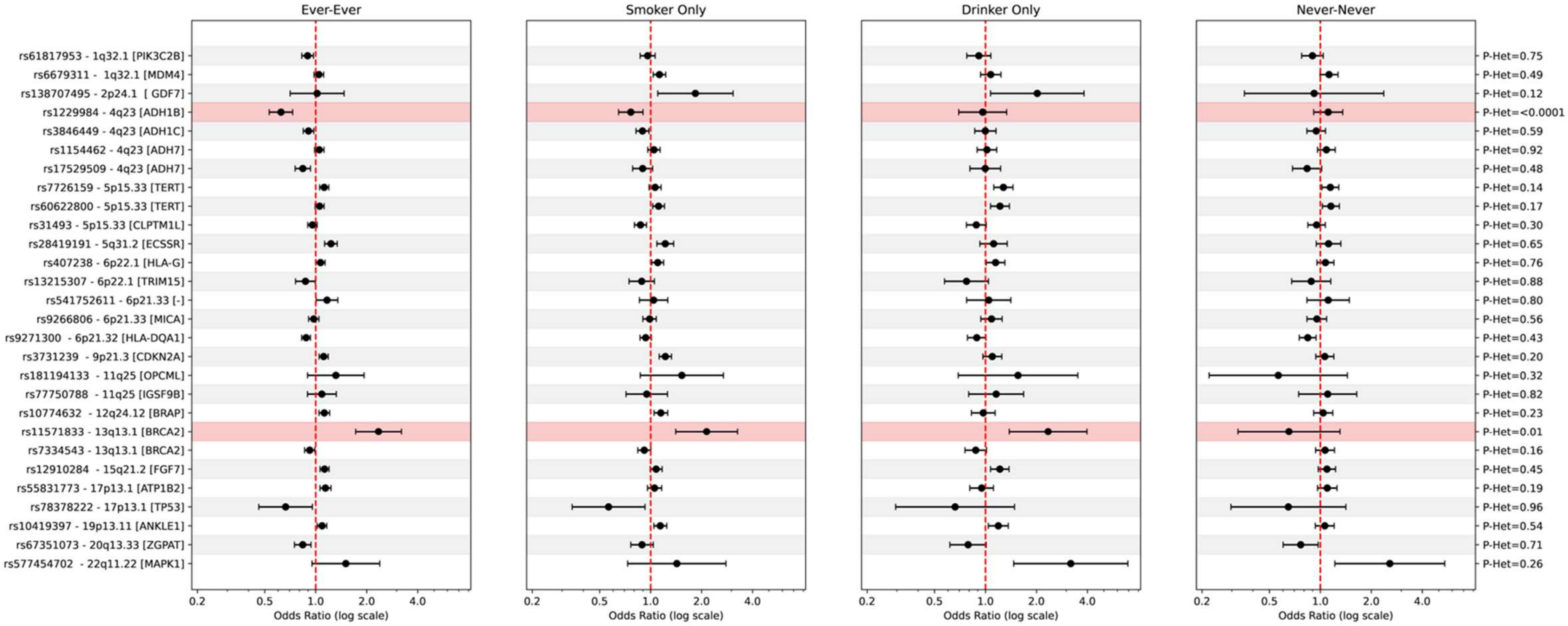

Supplementary Figure 9. Gene-environment interactions with alcohol and smoking. Effect estimates are stratified by combined smoking and drinking status (Never Smoker–Never Drinker, Smoker Only, Drinker Only, and Ever Smoker–Ever Drinker) for the top hit independent variants identified in both overall and subsite-specific GWAS meta-analyses. The left y-axis displays the rsID, cytoband, and nearest gene, while the right y-axis shows the p-value for heterogeneity across the four groups. Variants with a heterogeneity p-value < 0.05 are highlighted in red. For correlated variants, only the variant preferentially identified in the subsite-specific analysis is depicted; otherwise, a single representative variant is shown. Source data are provided as a Source Data file.

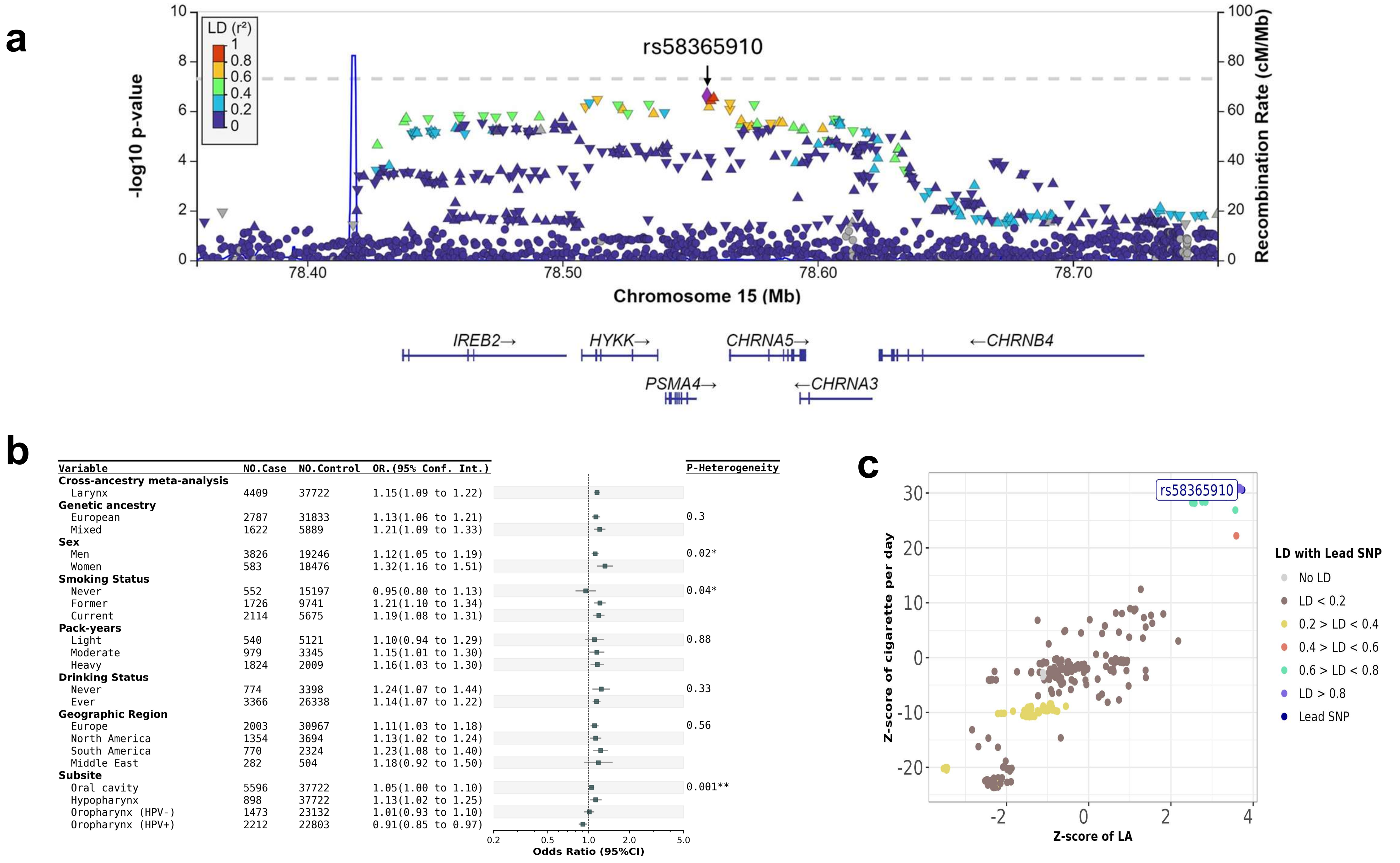

Supplementary Figure 10. rs58365910 shows a suggestive association with laryngeal cancer at the 15q25 locus. a) Regional plot of rs58365910, an intergenic variant mapped to *CHRNA5/PSMA4, showed a* suggestive association with laryngeal cancer (p_{meta}=2.53x10^{-7}). b) Forest plot of odds ratios for rs58365910 stratified by sex, smoking status, pack-year, drinking status, and geographic region. c) rs58365910 was colocalized with cigarette-smoked per day (CPD) (PP4 score=0.89).

a **Cross-ancestry (all sites combined)**

Chr6:33046667

Variable	NO.Case	NO.Control	OR(95% Conf. Int.)	P-Heterogeneity
**Cross-ancestry meta-analysis**				
All sites combined	18865	36207	1.10(1.07 to 1.14)	
**Genetic ancestry**				
Admixed	4300	4520	1.06(0.98 to 1.13)	0.63
African	403	280	1.13(0.87 to 1.47)	
European	13360	30277	1.12(1.08 to 1.17)	
Middle East	491	479	1.07(0.87 to 1.32)	
South Asian	311	651	1.04(0.80 to 1.36)	
**Drinking Status**				
Ever	12722	30490	1.09(1.05 to 1.14)	0.99
Never	2855	3049	1.09(0.99 to 1.20)	
**Sex**				
Female	4136	16753	1.07(0.97 to 1.19)	0.59
Male	14729	19454	1.11(1.06 to 1.15)	
**Smoking Status**				
Current	6854	5055	1.07(0.98 to 1.17)	0.76
Former	5749	11655	1.11(1.05 to 1.18)	
Never	3779	17626	1.09(1.00 to 1.20)	
**Subsite**				
Hypopharynx	798	34797	1.07(0.95 to 1.21)	0.01
Larynx	4357	35556	1.08(1.02 to 1.15)	
Oral cavity	5536	36207	1.04(0.98 to 1.09)	
Oropharynx (HPV-)	1452	35077	1.17(1.06 to 1.28)	
Oropharynx (HPV+)	2182	34797	1.22(1.12 to 1.33)	
**GeographicRegion**				
Europe	7716	29343	1.04(0.89 to 1.22)	0.76
NorthAmerica	7393	3817	1.11(1.04 to 1.19)	
SouthAmerica	2973	2294	1.09(1.00 to 1.19)	

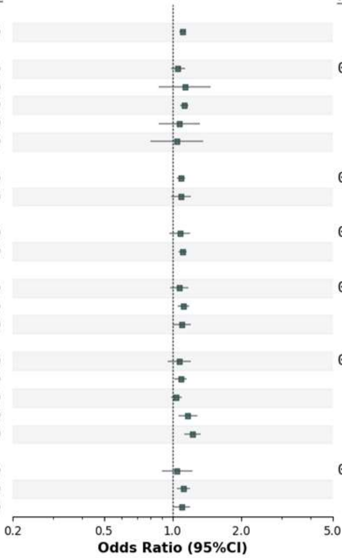

rs28360051

Variable	NO.Case	NO.Control	OR(95% Conf. Int.)	P-Heterogeneity
**Cross-ancestry meta-analysis**				
All sites combined	18865	36207	1.23(1.14 to 1.33)	
**Genetic ancestry**				
Admixed	4300	4520	1.13(0.93 to 1.37)	0.56
African	403	280	1.00(0.39 to 2.56)	
European	13360	30277	1.26(1.15 to 1.37)	
**Drinking Status**				
Ever	12722	30490	1.14(0.95 to 1.37)	0.78
Never	2855	3049	1.20(0.90 to 1.58)	
**Sex**				
Female	4136	16753	1.29(1.01 to 1.66)	0.49
Male	14729	19454	1.15(0.92 to 1.44)	
**Smoking Status**				
Current	6854	5055	1.22(1.02 to 1.47)	0.41
Former	5749	11655	1.11(0.86 to 1.43)	
Never	3779	17626	1.34(1.16 to 1.54)	
**Subsite**				
Hypopharynx	798	34797	1.02(0.47 to 2.25)	0.01
Larynx	4357	35556	1.06(0.91 to 1.22)	
Oral cavity	5536	36207	1.11(0.98 to 1.26)	
Oropharynx (HPV-)	1452	35077	1.04(0.68 to 1.59)	
Oropharynx (HPV+)	2182	34797	1.54(1.31 to 1.80)	
**GeographicRegion**				
Europe	7716	29343	1.29(1.16 to 1.42)	0.25
NorthAmerica	7393	3817	1.54(0.93 to 2.53)	
SouthAmerica	2973	2294	0.79(0.42 to 1.49)	

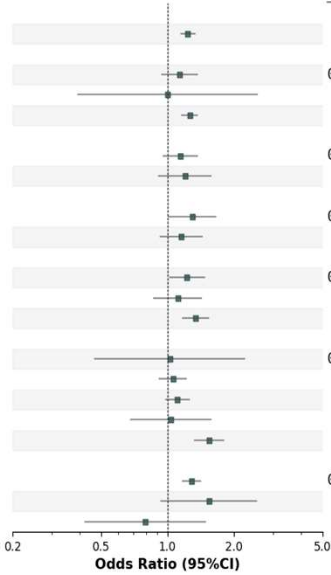

b Cross-ancestry (HPV(-) oropharynx)

rs1131212

Variable	NO.Case	NO.Control	OR(95% Conf. Int.)	P-Heterogeneity
**Cross-ancestry meta-analysis**				
Oropharynx (HPV-)	1452	35077	1.33(1.19 to 1.48)	
**Genetic ancestry**				
Admixed	453	4520	1.33(1.06 to 1.66)	0.62
African	65	280	1.03(0.61 to 1.75)	
European	934	30277	1.35(1.18 to 1.54)	
**Drinking Status**				
Ever	1156	30134	1.31(1.08 to 1.58)	
**Sex**				
Female	298	16273	1.61(1.09 to 2.39)	0.29
Male	1140	18708	1.29(1.14 to 1.46)	
**Smoking Status**				
Current	785	4850	1.30(1.10 to 1.54)	0.63
Former	411	11432	1.39(1.10 to 1.76)	
**Subsite**				
Hypopharynx	798	34797	1.18(1.03 to 1.36)	0.00
Larynx	4357	35556	1.08(1.01 to 1.16)	
Oral cavity	5536	36207	1.15(1.08 to 1.22)	
Oropharynx (HPV-)	1452	35077	1.33(1.19 to 1.48)	
Oropharynx (HPV+)	2182	34797	0.94(0.87 to 1.03)	
**GeographicRegion**				
Europe	679	28929	1.46(1.24 to 1.71)	0.69
NorthAmerica	407	3817	1.31(0.81 to 2.10)	
SouthAmerica	352	2234	7.53(0.10 to 544.21)	

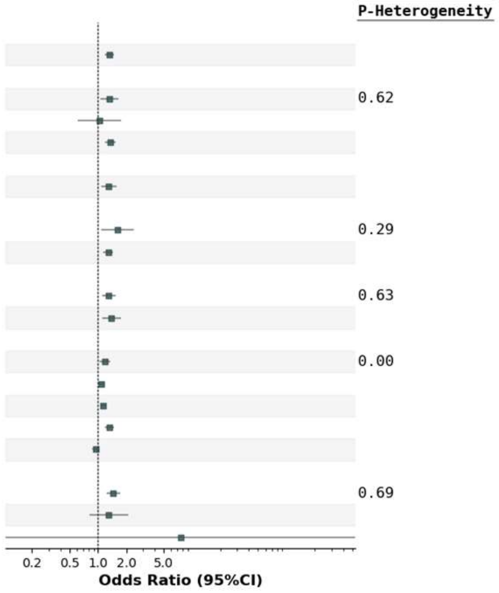

c Cross-ancestry (HPV(+) oropharynx)

DRB1 37Asn/Ser

Variable	NO.Case	NO.Control	OR(95% Conf. Int.)	P-Heterogeneity
**Cross-ancestry meta-analysis**				
Oropharynx (HPV+)	2182	34797	0.68(0.63 to 0.73)	
**Genetic ancestry**				
Admixed	222	4520	0.73(0.59 to 0.91)	0.43
European	1960	30277	0.67(0.62 to 0.73)	
**Drinking Status**				
Ever	1517	29911	0.69(0.63 to 0.76)	0.88
Never	417	2291	0.67(0.51 to 0.89)	
**Sex**				
Female	367	16273	0.61(0.45 to 0.84)	0.41
Male	1815	18524	0.70(0.64 to 0.76)	
**Smoking Status**				
Current	322	4778	0.57(0.42 to 0.78)	0.35
Former	987	11432	0.70(0.54 to 0.90)	
Never	869	16855	0.73(0.64 to 0.82)	
**Subsite**				
Hypopharynx	872	35556	0.95(0.79 to 1.16)	0.00
Larynx	4374	36207	1.04(0.92 to 1.16)	
Oral cavity	5536	36207	0.97(0.92 to 1.02)	
Oropharynx (HPV-)	1452	35077	0.85(0.75 to 0.97)	
Oropharynx (HPV+)	2182	34797	0.68(0.63 to 0.73)	
**GeographicRegion**				
Europe	1086	28929	0.70(0.48 to 1.03)	0.96
NorthAmerica	989	3633	0.71(0.62 to 0.80)	

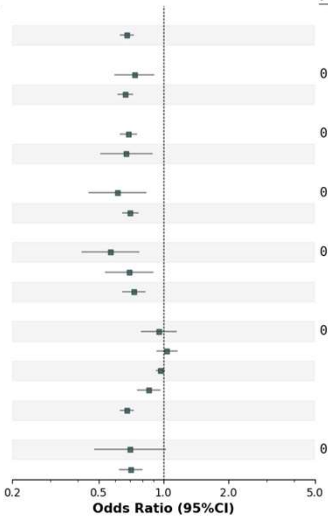

rs4143334

Variable	NO.Case	NO.Control	OR(95% Conf. Int.)	P-Heterogeneity
**Cross-ancestry meta-analysis**				
Oropharynx (HPV+)	2178	34797	1.89(1.51 to 2.35)	
**Genetic ancestry**				
Admixed	222	4520	1.55(0.81 to 2.96)	0.53
European	1956	30277	1.93(1.53 to 2.45)	
**Drinking Status**				
Ever	1514	29911	1.96(1.50 to 2.56)	0.74
Never	416	2291	1.76(0.96 to 3.22)	
**Sex**				
Female	367	16273	1.95(1.19 to 3.19)	0.83
Male	1811	18524	1.84(1.44 to 2.36)	
**Smoking Status**				
Current	322	4778	1.04(0.53 to 2.03)	0.25
Former	985	11432	1.84(0.73 to 4.58)	
Never	867	16855	2.15(1.23 to 3.75)	
**Subsite**				
Hypopharynx	872	35556	1.08(0.76 to 1.53)	0.00
Larynx	4371	36207	0.96(0.80 to 1.15)	
Oral cavity	5534	36207	0.95(0.80 to 1.13)	
Oropharynx (HPV-)	1449	35077	0.79(0.56 to 1.10)	
Oropharynx (HPV+)	2178	34797	1.89(1.51 to 2.35)	
**GeographicRegion**				
Europe	1082	28929	1.91(1.40 to 2.60)	0.85
NorthAmerica	989	3633	1.83(1.27 to 2.63)	

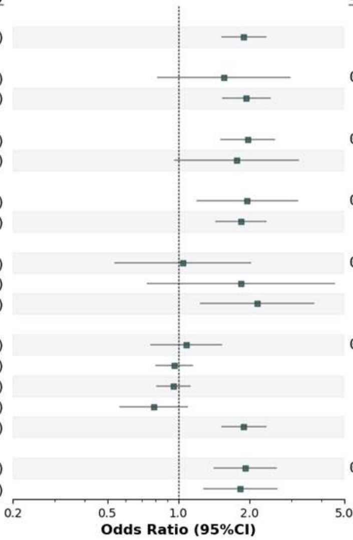

DRB1 233Thr

Variable	NO.Case	NO.Control	OR(95% Conf. Int.)	P-Heterogeneity
**Cross-ancestry meta-analysis**				
Oropharynx (HPV+)	2182	34797	1.27(1.17 to 1.38)	
**Genetic ancestry**				
Admixed	222	4520	1.16(0.94 to 1.45)	0.39
European	1960	30277	1.29(1.18 to 1.41)	
**Drinking Status**				
Ever	1517	29911	1.20(0.99 to 1.47)	0.54
Never	417	2291	1.40(0.89 to 2.21)	
**Sex**				
Female	367	16273	1.26(1.04 to 1.51)	0.89
Male	1815	18524	1.27(1.16 to 1.39)	
**Smoking Status**				
Current	322	4778	1.11(0.88 to 1.39)	0.66
Former	987	11432	1.26(0.96 to 1.65)	
Never	869	16855	1.25(1.09 to 1.42)	
**Subsite**				
Hypopharynx	872	35556	0.96(0.86 to 1.07)	0.00
Larynx	4374	36207	0.98(0.92 to 1.03)	
Oral cavity	5536	36207	1.00(0.92 to 1.09)	
Oropharynx (HPV-)	1452	35077	0.93(0.80 to 1.07)	
Oropharynx (HPV+)	2182	34797	1.27(1.17 to 1.38)	
**GeographicRegion**				
Europe	1086	28929	1.35(1.04 to 1.76)	0.32
NorthAmerica	989	3633	1.17(1.03 to 1.33)	

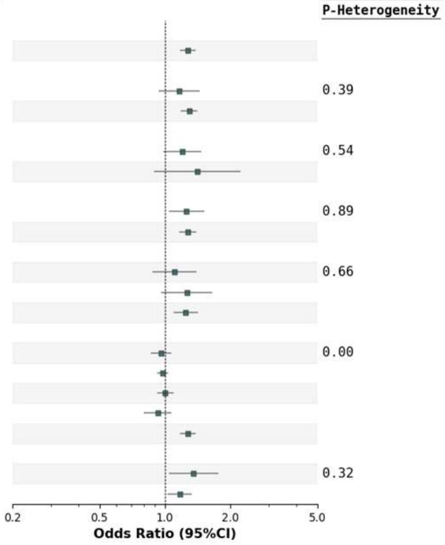

B 67Cys/Ser/Tyr

Variable	NO.Case	NO.Control	OR(95% Conf. Int.)	P-Heterogeneity
**Cross-ancestry meta-analysis**				
Oropharynx (HPV+)	2182	34797	0.81(0.74 to 0.88)	
**Genetic ancestry**				
Admixed	222	4520	0.83(0.66 to 1.04)	0.85
European	1960	30277	0.81(0.73 to 0.88)	
**Drinking Status**				
Ever	1517	29911	0.78(0.71 to 0.87)	0.50
Never	417	2291	0.85(0.68 to 1.06)	
**Sex**				
Female	367	16273	0.81(0.66 to 0.98)	0.95
Male	1815	18524	0.81(0.74 to 0.89)	
**Smoking Status**				
Current	322	4778	0.82(0.65 to 1.02)	0.72
Former	987	11432	0.76(0.67 to 0.87)	
Never	869	16855	0.82(0.72 to 0.94)	
**Subsite**				
Hypopharynx	872	35556	0.90(0.80 to 1.02)	0.00
Larynx	4374	36207	0.98(0.92 to 1.04)	
Oral cavity	5536	36207	0.87(0.82 to 0.92)	
Oropharynx (HPV-)	1452	35077	0.95(0.82 to 1.09)	
Oropharynx (HPV+)	2182	34797	0.81(0.74 to 0.88)	
**GeographicRegion**				
Europe	1086	28929	0.79(0.70 to 0.89)	0.76
NorthAmerica	989	3633	0.81(0.68 to 0.97)	

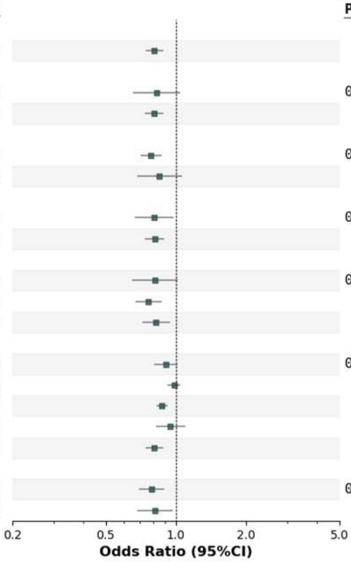

d **Admixed (all sites combined)**

rs1536036

Variable	NO.Case	NO.Control	OR(95% Conf. Int.)	P-Heterogeneity
**Cross-ancestry meta-analysis**				
All sites combined	18865	36207	0.99(0.88 to 1.12)	
**Genetic ancestry**				
Admixed	4300	4520	0.85(0.80 to 0.91)	0.00
African	403	280	1.42(1.12 to 1.79)	
European	13360	30277	0.99(0.95 to 1.02)	
Middle East	491	479	0.89(0.73 to 1.09)	
South Asian	311	651	1.09(0.86 to 1.39)	
**Drinking Status**				
Ever	12722	30490	0.98(0.86 to 1.12)	0.85
Never	2953	3106	0.96(0.82 to 1.13)	
**Sex**				
Female	4136	16753	0.97(0.87 to 1.08)	0.73
Male	14729	19454	1.00(0.86 to 1.15)	
**Smoking Status**				
Former	5749	11655	0.97(0.77 to 1.24)	0.92
Never	3810	17741	0.97(0.91 to 1.02)	
**Subsite**				
Hypopharynx	872	35556	0.93(0.77 to 1.12)	0.89
Larynx	4374	36207	0.95(0.81 to 1.11)	
Oral cavity	5536	36207	0.99(0.89 to 1.11)	
Oropharynx (HPV-)	1452	35077	1.01(0.78 to 1.31)	
Oropharynx (HPV+)	2182	34797	0.92(0.82 to 1.03)	

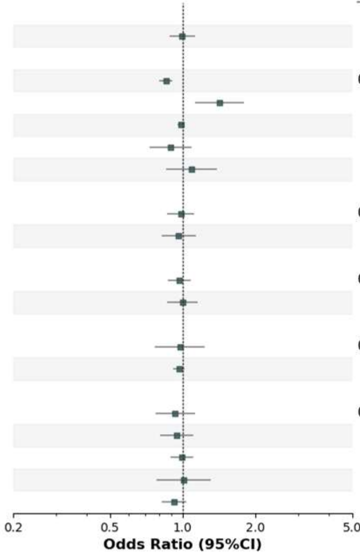

e **European (Oral cavity)**

DRB1 74Ala/Leu/Del

Variable	NO.Case	NO.Control	OR(95% Conf. Int.)	P-Heterogeneity
**Cross-ancestry meta-analysis**				
OralCavity	5536	36207	0.86(0.75 to 1.00)	
**Genetic ancestry**				
Admixed	1574	4520	1.02(0.92 to 1.12)	0.00
African	122	280	0.72(0.50 to 1.04)	
European	3463	30277	0.82(0.77 to 0.87)	
Middle East	145	479	0.90(0.65 to 1.24)	
South Asian	232	651	0.77(0.56 to 1.04)	
**Drinking Status**				
Ever	4040	30490	0.88(0.75 to 1.05)	0.65
Never	1216	3106	0.84(0.71 to 0.99)	
**Sex**				
Female	1805	16753	0.89(0.71 to 1.12)	0.78
Male	3731	19454	0.86(0.74 to 1.00)	
**Smoking Status**				
Former	1556	11589	0.85(0.70 to 1.03)	0.86
Never	1483	17626	0.88(0.77 to 1.01)	
**Subsite**				
Hypopharynx	872	35556	0.84(0.76 to 0.94)	0.51
Larynx	4374	36207	0.89(0.76 to 1.04)	
Oral cavity	5536	36207	0.86(0.75 to 1.00)	
Oropharynx (HPV-)	1452	35077	0.95(0.81 to 1.10)	
Oropharynx (HPV+)	2182	34797	0.82(0.76 to 0.88)	
**GeographicRegion**				
Europe	2269	28929	0.91(0.69 to 1.20)	0.79
NorthAmerica	1719	3817	0.82(0.66 to 1.02)	
SouthAmerica	1169	2294	0.93(0.64 to 1.35)	

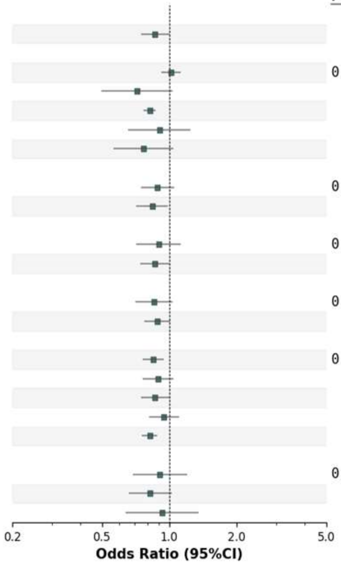

rs9267280

Variable	NO.Case	NO.Control	OR(95% Conf. Int.)	P-Heterogeneity
**Cross-ancestry meta-analysis**				
OralCavity	5536	36207	1.17(0.94 to 1.45)	
**Genetic ancestry**				
Admixed	1574	4520	0.95(0.82 to 1.11)	0.01
African	122	280	1.44(0.84 to 2.46)	
European	3463	30277	1.32(1.19 to 1.47)	
Middle East	145	479	0.98(0.60 to 1.62)	
South Asian	232	651	1.45(0.82 to 2.57)	
**Drinking Status**				
Ever	4040	30490	1.14(0.87 to 1.50)	0.78
Never	1216	3106	1.20(0.98 to 1.47)	
**Sex**				
Female	1805	16753	1.19(0.89 to 1.59)	0.90
Male	3731	19454	1.16(0.95 to 1.42)	
**Smoking Status**				
Former	1556	11589	1.13(0.78 to 1.64)	0.83
Never	1483	17626	1.23(1.05 to 1.43)	
**Subsite**				
Hypopharynx	872	35556	1.06(0.87 to 1.30)	0.66
Larynx	4374	36207	0.98(0.84 to 1.15)	
Oral cavity	5536	36207	1.17(0.94 to 1.45)	
Oropharynx (HPV-)	1452	35077	1.12(0.93 to 1.35)	
Oropharynx (HPV+)	2182	34797	1.01(0.89 to 1.16)	
**GeographicRegion**				
Europe	2269	28929	1.10(0.74 to 1.63)	0.72
NorthAmerica	1719	3817	1.23(0.68 to 2.21)	
SouthAmerica	1169	2294	0.99(0.83 to 1.18)	

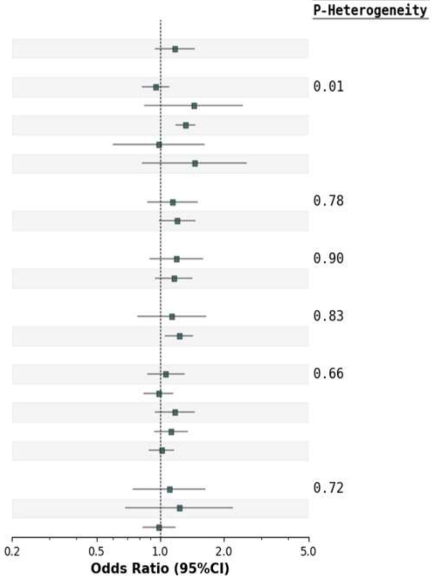

f **European (HPV(+) oropharynx)**

HLA-B*51:01

Variable	NO.Case	NO.Control	OR(95% Conf. Int.)	P-Heterogeneity
**Cross-ancestry meta-analysis**				
Oropharynx (HPV+)	2182	34797	1.37(0.69 to 2.72)	
**Genetic ancestry**				
Admixed	222	4520	0.94(0.61 to 1.45)	0.00
European	1960	30277	1.90(1.55 to 2.31)	
**Drinking Status**				
Ever	1517	29911	1.38(0.61 to 3.10)	0.78
Never	437	2296	1.57(0.99 to 2.49)	
**Sex**				
Female	367	16273	1.54(0.40 to 5.86)	0.87
Male	1815	18524	1.36(0.80 to 2.34)	
**Smoking Status**				
Former	987	11432	1.30(0.58 to 2.95)	0.68
Never	869	16855	1.87(1.39 to 2.51)	
**Subsite**				
Hypopharynx	872	35556	1.08(0.85 to 1.37)	0.77
Larynx	4374	36207	1.10(0.97 to 1.24)	
Oral cavity	5536	36207	1.20(1.08 to 1.34)	
Oropharynx (HPV-)	1452	35077	1.15(0.88 to 1.49)	
Oropharynx (HPV+)	2182	34797	1.37(0.69 to 2.72)	
**GeographicRegion**				
Europe	1086	28929	1.88(1.23 to 2.89)	0.67
NorthAmerica	989	3633	1.69(1.27 to 2.25)	

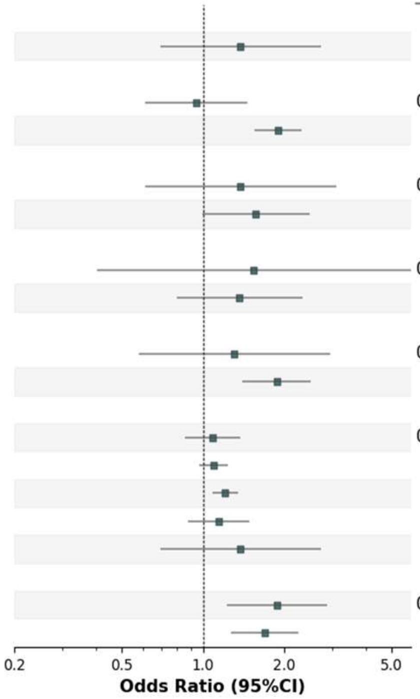

Supplementary Figure 11. Forest plots of novel variants from HLA fine-mapping analyses. For each independent novel top hit identified in the cross-ancestry HLA fine-mapping and ancestry-specific, analyses were stratified by sex, smoking status, drinking status, geographic region, and cancer subsites. Forest plots of variants identified by cancer subsite: a) top hit variants identified in all sites combined, b) variants identified in the HPV- oropharynx, c) variants identified in HPV+ oropharynx, d) variants identified in all sites combined specific to admixed population, e) variants identified in oral cavity specific to European population, and f) variants identified in HPV+ oropharynx specific to European population.

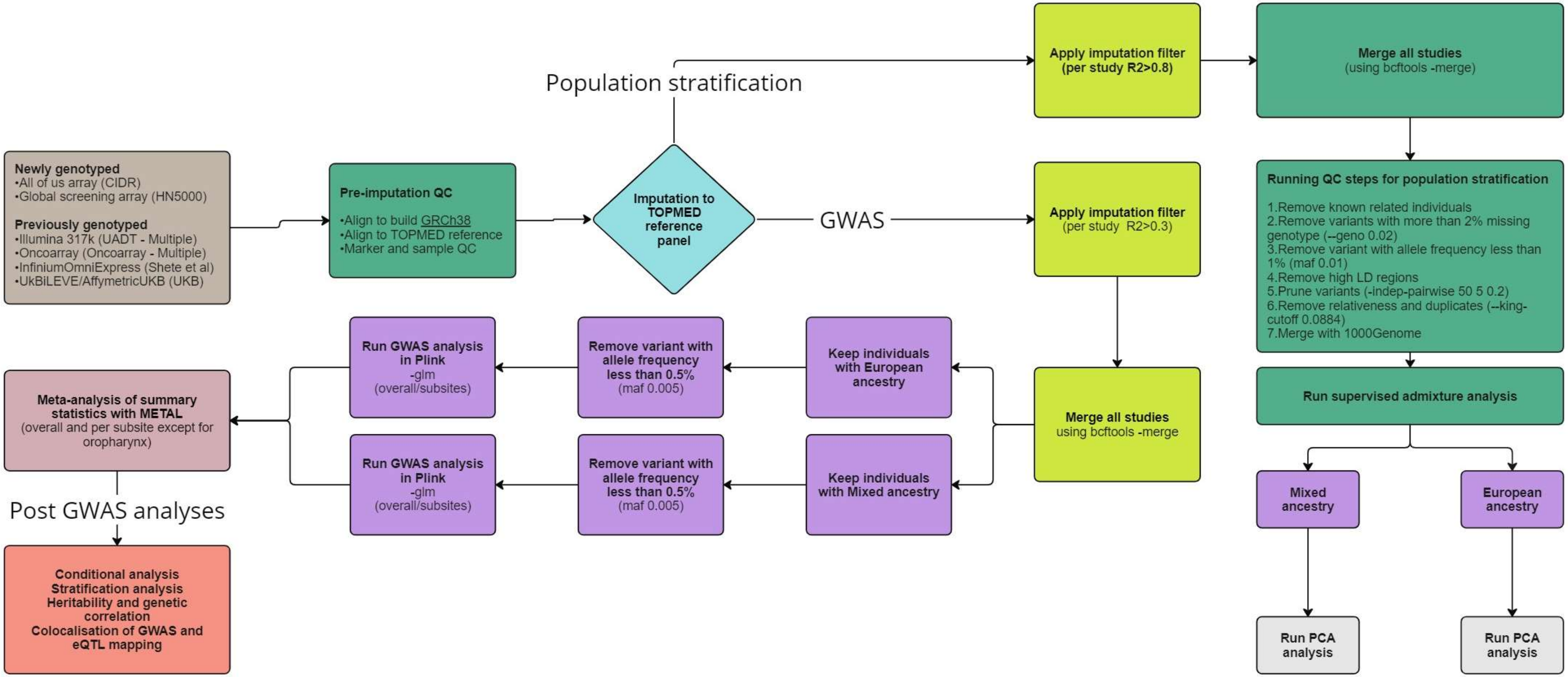

Supplementary Figure 12. Flow diagram showing steps of genetic data curation and quality control (QC), imputation and analysis for GWAS.
CIDR = Centre for Inherited Disease Research, UADT = Upper aerodigestive tract GWAS paper (McKay et al. 2011), UKB = UK Biobank, MAF = Minor allele frequency, LD = Linkage disequilibrium, PCA = Principal component analysis, eQTL= Expression quantitative trait loci (Created by Miro).

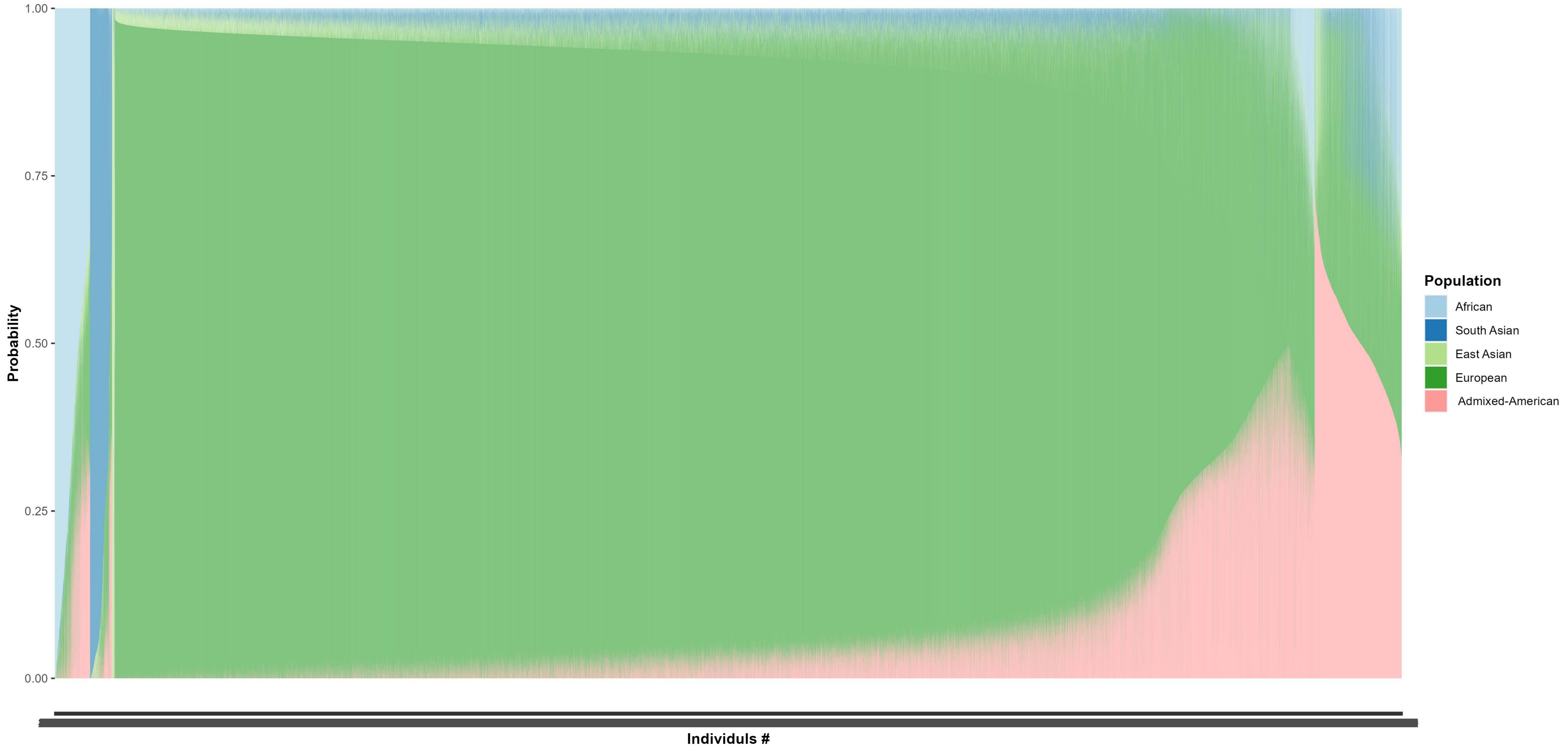

Supplementary Figure 13. Population stratification using supervised Admixture analysis. Supervised ADMIXTURE analysis was conducted on a dataset comprising 61,129 individuals, with the 1000 Genome super populations serving as the reference which includes 2504 individuals. Each point on the x-axis corresponds to an individual, while the y-axis indicates their ancestry proportion.

a

GWAS Group	Ancestry group	Case					Control
		Oral cavity	Larynx	Hypopharynx	Oropharynx	Other	
European	European	3511	2787	566	4134	2119	32914
Mixed	African	125	145	30	104	2	273
	South Asian	184	21	3	35	0	488
	East Asian	28	21	2	14	0	24
	Admixed American	6	8	0	7	0	30
	Admixed	1742	1427	297	1117	111	5128

b

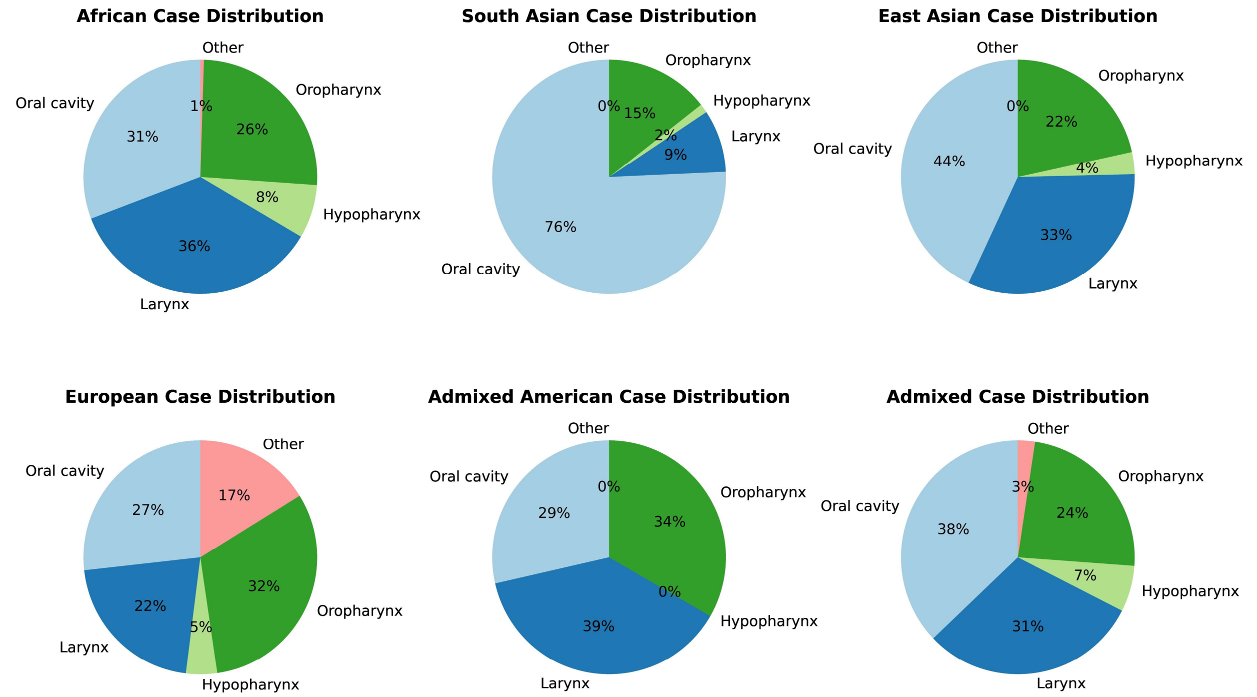

Supplementary Figure 14. Ancestral composition and case distribution across each ancestral group. a) The classification of individuals into various ancestral groups was based on a 70% threshold, distinguishing those with dominant ancestry (≥70%) from those with admixed ancestry (<70%). Due to the small sample size of non-European dominant ancestries (N = 1,561), these individuals were combined with the admixed cases and collectively referred to as the "Mixed" ancestry group. b) Distribution of cancer cases among different ancestral groups. The "Other" case group includes cases with unknown primary site, overlapping sites, Not Otherwise Specified (NOS), or unavailable data.

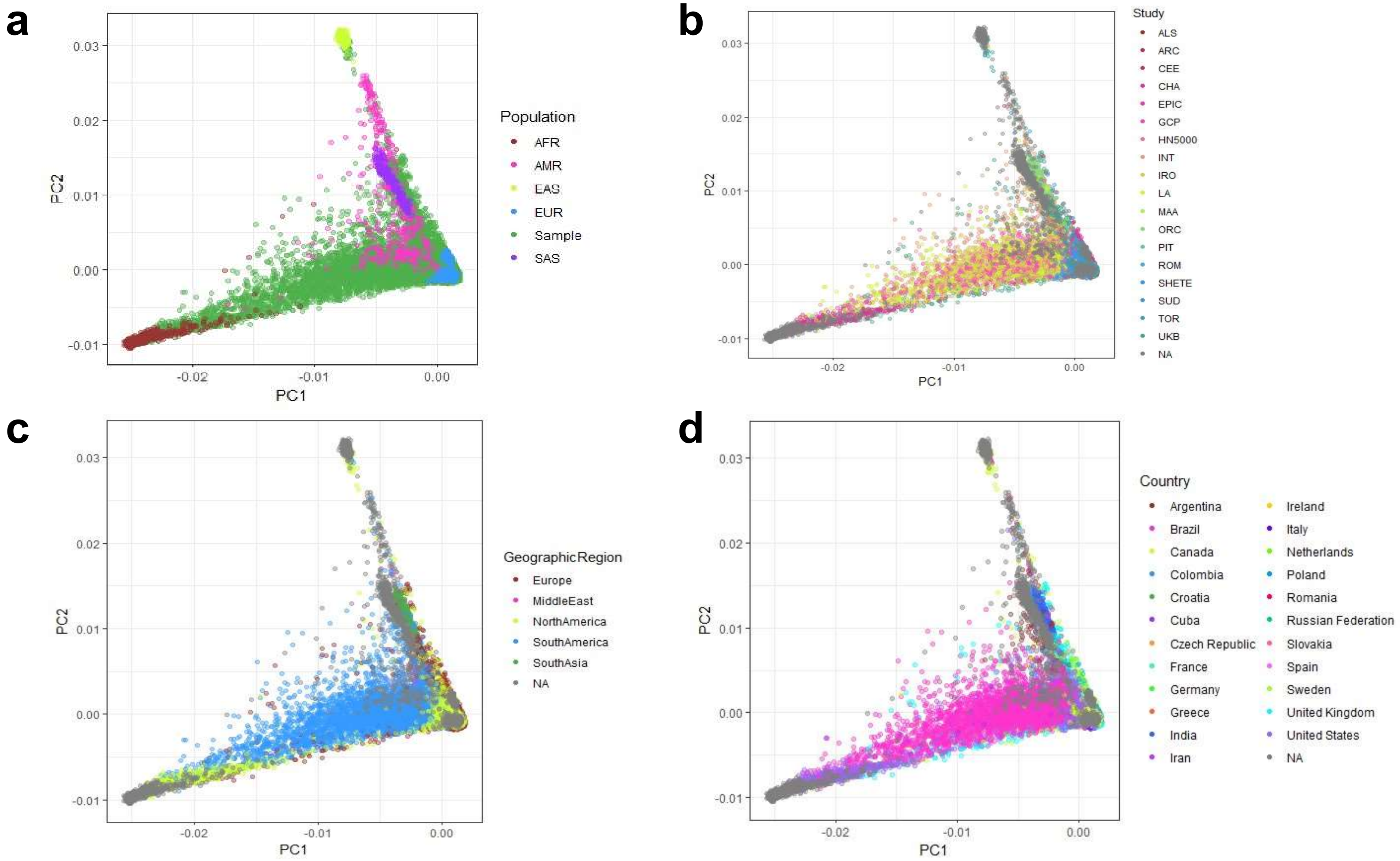

Supplementary Figure 15. Principle component analysis. Principal Component Analysis (PCA) was conducted on a dataset of 61,129 individuals, including 2,504 individuals from the 1000 Genomes Project, representing five super populations as a reference. The analysis is visualized using Principal Components 1 (PC1) and 2 (PC2). a) The PCA plot shows the study population (green) compared to the five super populations from the 1000 Genomes Project (AFR: African; AMR: Admixed American; EAS: East Asian; EUR: European; SAS: South Asian). b) The study population is grouped by the geographic region of recruitment. c) The population is categorized by the study they were involved in. d) The population is displayed based on country. The 1000 Genomes super populations are shown in grey in panels b, c, and d.

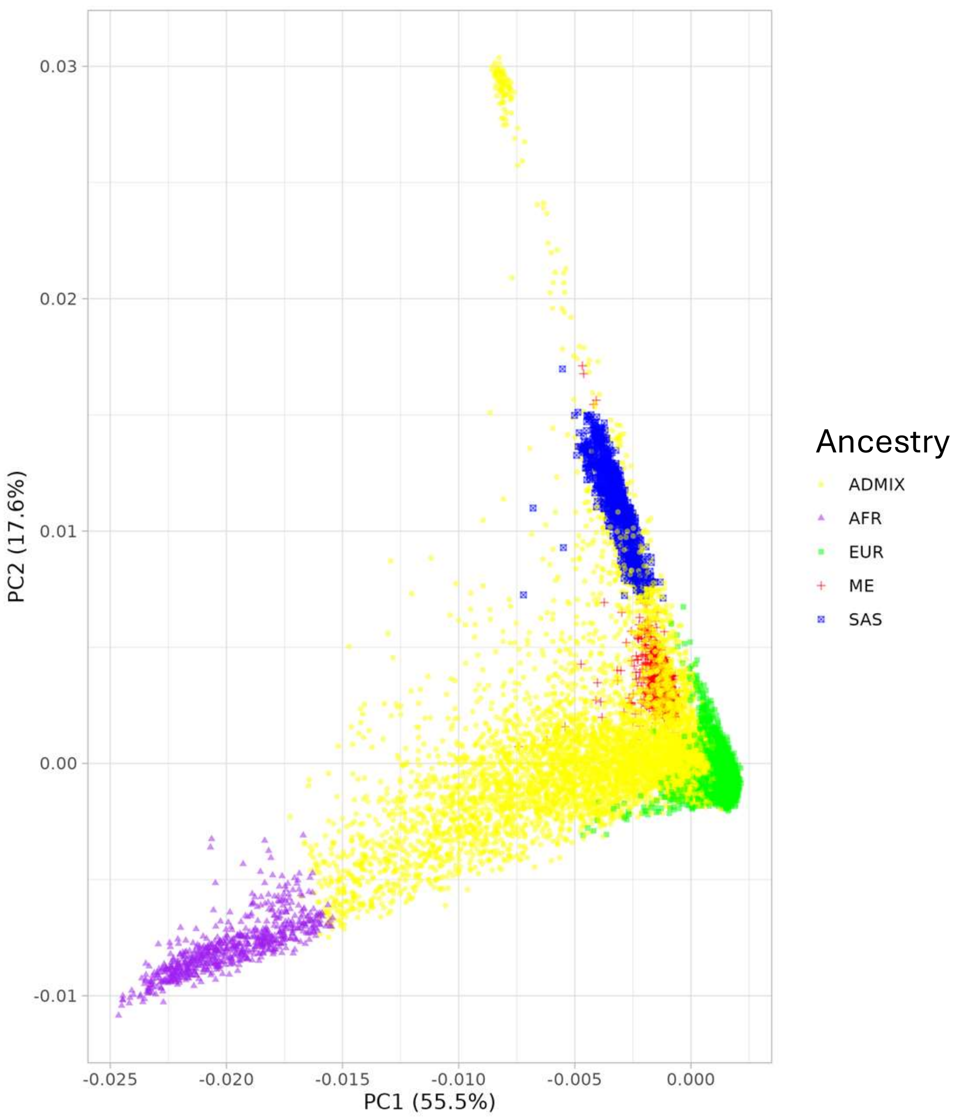

Supplementary Figure 16. PCA plot of population stratification using supervised Admixture analysis for HLA analysis. Principal Component Analysis (PCA) was conducted to represent five population ancestries. The analysis is visualized using Principal Components 1 (PC1) and 2 (PC2). AFR: African is coloured in purple; ADMIX in yellow; EUR: European in green; SAS: South Asian in blue and ME: Middle East in red.

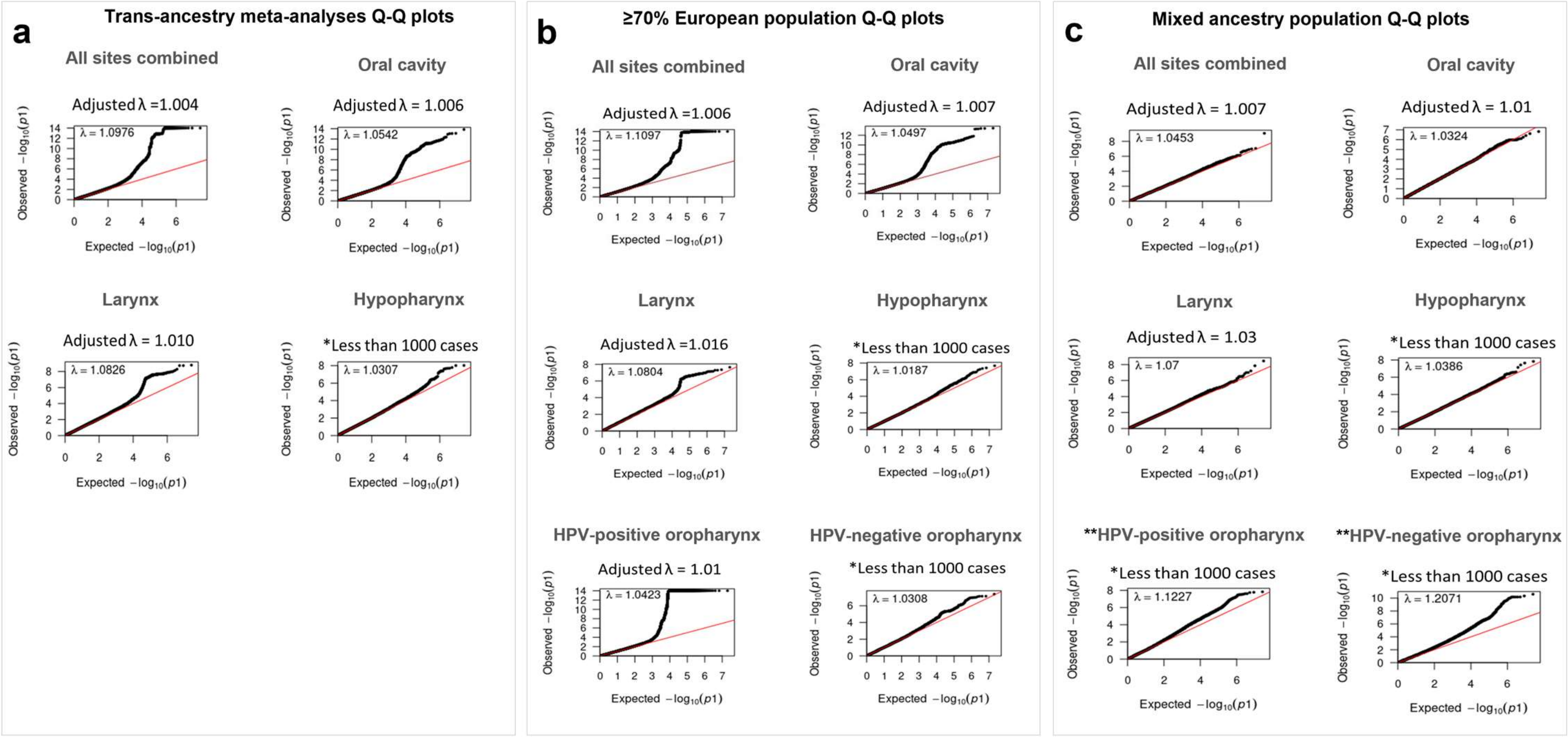

Supplementary Figure 17. Cross ancestry and population-specific quantile-quantile (Q-Q) plots. a) Q-Q plots from subsite analyses in cross-ancestry meta-analysis GWAS. b) Q-Q plots from GWAS with a predominant European sample representation (≥70%). c) Q-Q plots from analyses conducted on mixed ancestry populations. The red line represents the expected null distribution. "λ" = genomic inflation factor; "Adjusted λ" = genomic inflation factor for an equivalent study of 1,000 cases and 1,000 controls;

*For instances with fewer than 1,000 cases, only λ was reported.

**Since the Q-Q plot for HPV(+) and HPV(-) oropharyngeal cases in the mixed ancestry population showed inflation, the results were excluded from further analyses in this study.

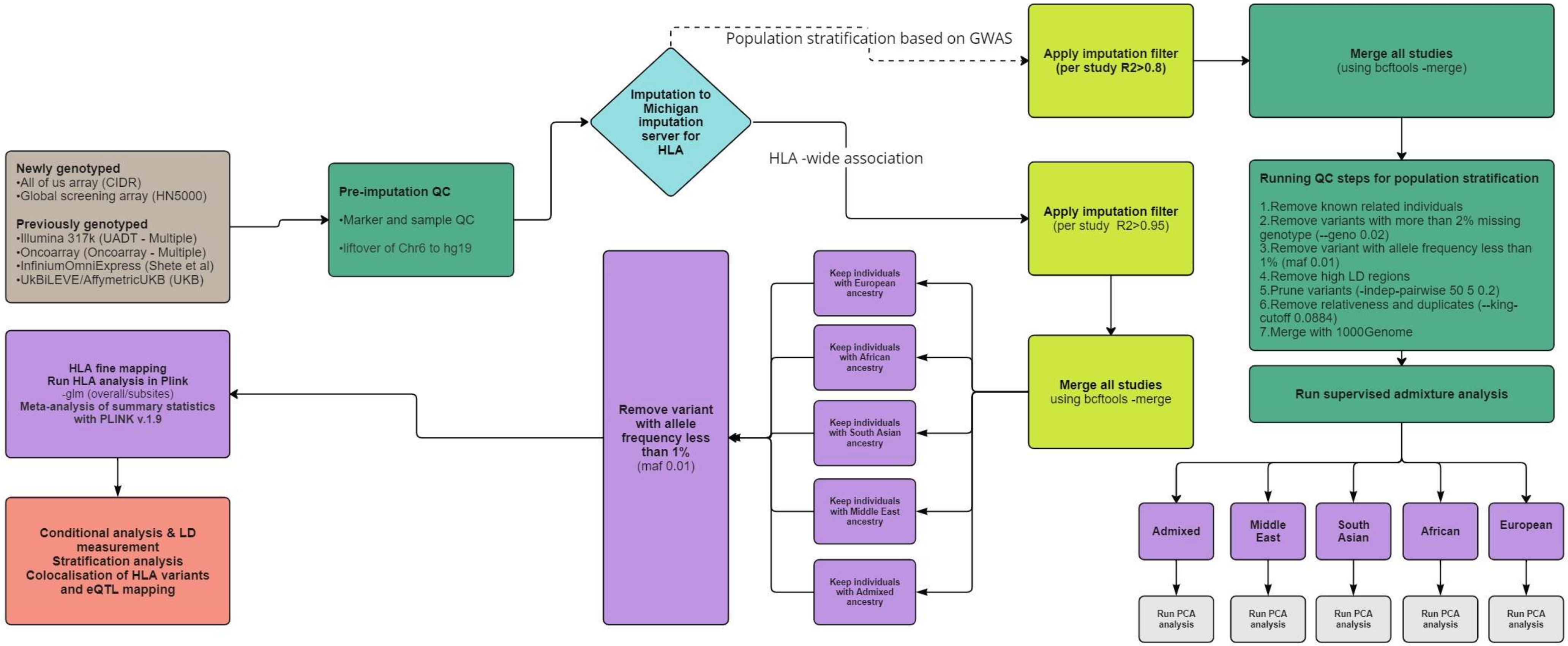

Supplementary Figure 18. Flow diagram showing steps of genetic data curation and quality control (QC), imputation and analysis for HLA fine mapping.
CIDR = Centre for Inherited Disease Research, UADT = Upper airway digestive tract GWAS paper (McKay et al. 2011), UKB = UK Biobank, MAF = Minor allele frequency, LD = Linkage disequilibrium, PCA = Principal component analysis.

Supplementary Note 1: HN5000 ALSPAC combined imputation notes

This note describes the ‘double imputation’ method used to imputed the HN5000 cases with previously genotyped ALSPAC controls. Given the two studies have been genotyped on different arrays, simply combining the genotyped variants and imputing would mean the majority of variants from both studies are dropped as there is limited overlap between arrays.

The strategy here uses 2 rounds of imputation, the first allows high quality imputed variants to be used alongside variants genotyped on only one of the arrays for the second round of imputation.

ALSPAC re-genotyped – 411 ALSPAC participants who had previously been genotyped were re-genotyped alongside the HN5000 cases (DNA from immortalised cell lines). This allows some sensitivity analyses to be conducted with cases and controls genotyped together on the same array (Figure S19).

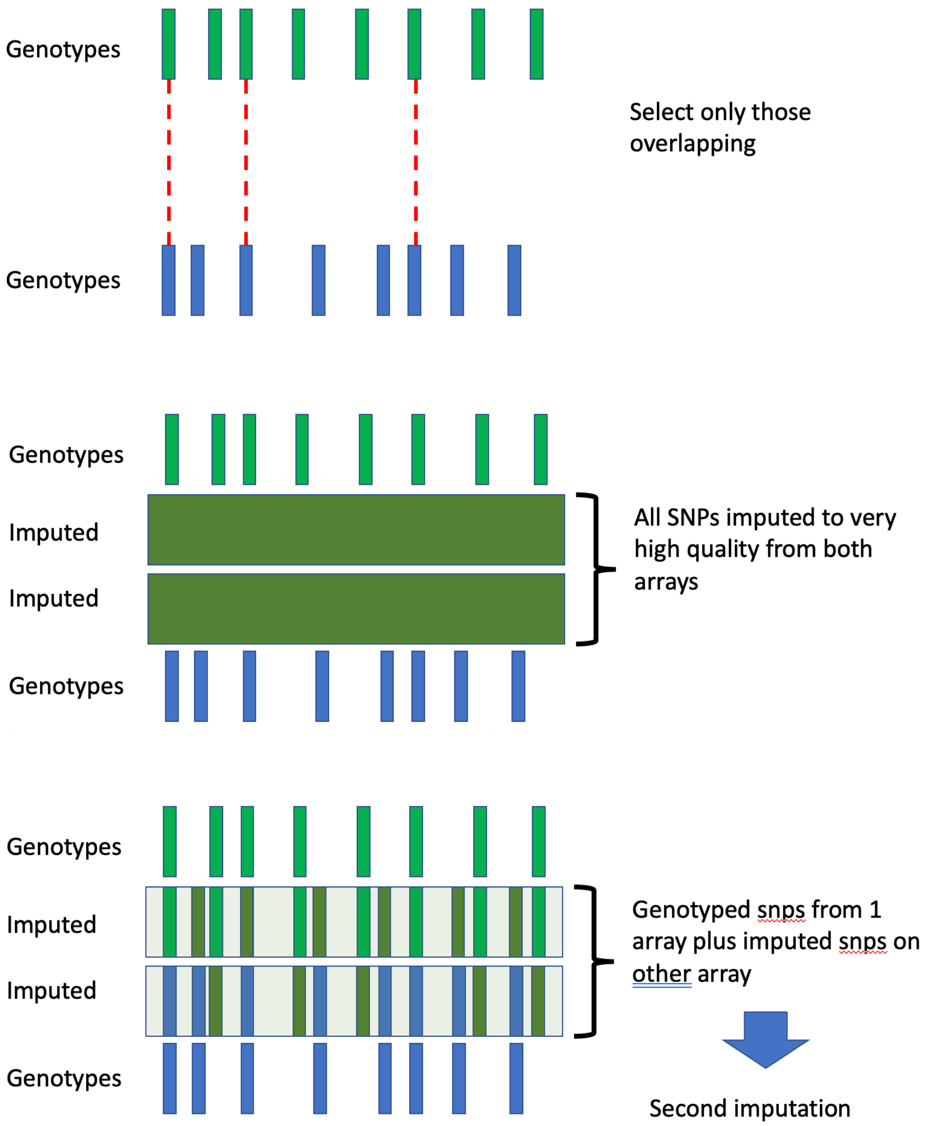

Supplementary Figure 19: Schematic detailing double imputation method.

1) Genotype calling from Intensity (IDat) files

- QC steps in GenomeStudio
- The GenomeStudio QC was run with HN5000 and ALSPAC (re-genotyped) samples combined.
- Sample QC
  - Import intensity files into GS
  - Cluster file from Ilumina: https://webdata.illumina.com/downloads/productfiles/global-screening-array/v2-0/gsa-24-v2-0-A1-cluster-file.zip
  - GSA manifest file from Illumina (build GRCh38): https://webdata.illumina.com/downloads/productfiles/global-screening-array/v2-0/infinium-global-screening-array-24-v2-0-a2-manifest-file-bpm.zip
  - Run sample calculations (to generate sample call rate)
  - Remove samples with <97% call rate (UK Biobank recommendation)
    - HN5000/ALSPAC – 71 samples removed
  - Recalculate SNP statistics
- SNP QC
  - Run SNP QC and zero SNPs not meeting thresholds (from Illumina technical note)
  - Sort SNP Table by Cluster Sep. zero SNPs with ≤0.3 (illumina recommendation)
    - HN5000/ALSPAC – 3865 removed
  - Sort SNP Table by call frequency (Call_Freq). Zero SNPs <0.97 (illumina rec)
    - HN5000/ALSPAC – 14504 removed
  - Sort SNP Table by AB R Mean, the mean normalized intensity (R) of the heterozygote cluster. This metric helps identify SNPs with low intensity data and has values increasing from 0. Zero SNPs ≤0.2
    - HN5000/ALSPAC – 38 removed
  - Sort SNP Table by AB T Mean, the mean of the normalized theta values of the heterozygote cluster. Zero SNPs ≤0.2
    - HN5000/ALSPAC –540 removed
- Update the sample statistics
- Sex mismatch
  - Add reported sex to Gender column
  - Estimate Gender – select all samples, right click select Estimate gender
  - Assess for mismatch and investigate possibility of mislabelled samples
    - HN5000/ALSPAC – 21 reported Female but GS reports Male
    - HN5000/ALSPAC – 18 reported Male but GS reports Female

2) Round 1 Imputation

Each study was imputed to TOPMED imputation panel following completing QC steps

3) Combine HN5000 and ALSPAC ready for round 2 imputation.

Aim: To create a set of variants from both studies which are either genotyped or imputed to a very high quality but also are informative for imputation. To ensure the variants are informative, the variants selected must be included on at least one of the arrays.

NB. In previous attempts, only selecting variants which are of high quality imputation does not ensure imputation informative variants are included in the combination - this resulted in poor quality round 2 imputation.

Following round 1 imputation:

- HN5000
  - 480,496 genotyped variants (a)
  - 307,174,027 imputed variants
- ALSPAC
  - 451,189 genotyped variants (a)
  - 306,899,501 imputed variants

Steps to select variants:

- Identify variants genotyped on both arrays
  - 94,289
- Identify variants genotyped in one study and imputed in the other
  - Imputed in HN5000 and genotyped in ALSPAC = 356,900
  - Imputed in ALSPAC and genotyped in HN5000 = 386,175
- Of these, identify those with high quality imputation (Rsq>0.90)
  - Imputed in HN5000 and genotyped in ALSPAC = 330,272 (b)
  - Imputed in ALSPAC and genotyped in HN5000 = 300,315 (b)
- Genotyped or high-quality imputation and on the other array (a + b)
  - HN5000 = 810,768
  - ALSPAC = 751,504
- Overlapping in both studies
  - 724,876
- Select these variants from both studies
- Combine the two studies
  - Bcftools merge –force-samples
- Convert imputed variants to 'Best Guess' genotypes ready for imputation
  - bcftools annotate -x FOMAT

Check imputation quality in the 2 studies of the overlapping variants (Figure S20)

- High quality and similar

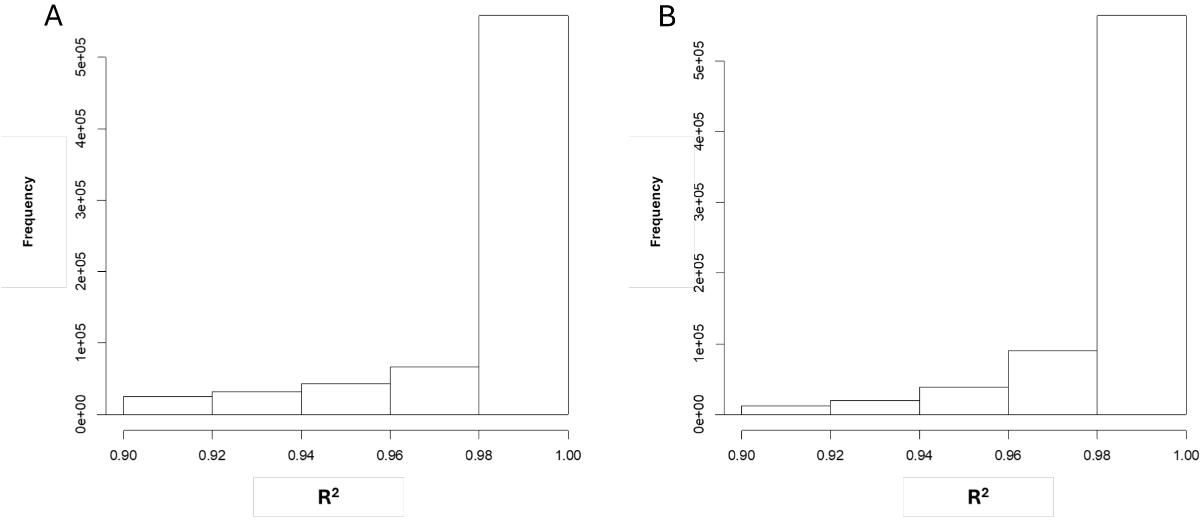

Supplementary Figure 20: Distribution of variants in imputation quality (R^2) bins in (A) ALSPAC and (B) HN5000.

4) Round 2 imputation

The combined studied are then imputed together to TOPMED

- Allele frequency r2 between uploaded samples and reference panel = 0.942

Supplementary Table 1. Technical validation of *TP53 variant* rs78378222 using Taqman assay

		Imputation genotype		
		T/T	G/T	G/G
**Taqman assay genotype**	G/G	0	1	0
	G/T	1	32	0
	T/T	2336	0	0

Technical validation was performed in 2,370 samples using Taqman assays for the rare *TP53* variant. Concordance was 99.9%
