# Supplementary material for: Cross-ancestral GWAS identifies 29 variants across head and neck cancer subsites
Source: Nat Commun. 2025 Oct 2;16:8787. doi: 10.1038/s41467-025-63842-z (PMC12491539; doi:10.1038/s41467-025-63842-z)
Supplement: Supplementary file 3 — Description of Additional Supplementary Files [file 41467_2025_63842_MOESM3_ESM.pdf]

Description of Additional Supplementary Files

Supplementary Data 1. Characterization of studies included in ancestry-specific and meta-analyses gwas.

Supplementary Data 2. Epidemiological and clinical characteristics of individuals included in this study.

Supplementary Data 3. Summary of genetic variants identified in European and Mixed groups through gwas and meta-analysis.

Supplementary Data 4. Description of independent genome-wide significant variants identified in ancestry specific and meta-analysis gwas analyses (in all sites combined and subsite specific).

Supplementary Data 5. Functional annotation of independent top-hit variants identified in cross-ancestry, European, and Mixed-ancestry gwas analyses.

Supplementary Data 6. Genetic heritability of head and neck cancer and subsites.

Supplementary Data 7. Colocalization analysis of significant SNPs.

Supplementary Data 8. Conditional analysis of HLA lead variants in head and neck cancer overall and each subsites.

Supplementary Data 9. Linkage disequilibrium measurement between HLA lead variants.

Supplementary Data 10. Summary of genetic variants identified across ancestry-specific and meta-analysis of HLA-fine mapping in all sites combined and subsite specific.

Supplementary Data 11. Description of lead variants from HLA fine-mapping analysis.

Supplementary Data 12. Association signal of novel amino acid/4digit allele highly linked to novel lead significant variants.

Supplementary Data 13. Power calculations.

Supplementary Data 14. Quality control steps for each genotyping array before imputation at variant and individual levels.

Supplementary Data 15. Mean and standard deviation of minor allele frequency across all imputation batches.

Supplementary Data 16. Association results of conditional analysis on cross-ancestry independent lead SNPs where there was a shared signal in a corresponding subsite.
