# Supplementary material for: Cross-ancestral GWAS identifies 29 variants across head and neck cancer subsites
Source: Nat Commun. 2025 Oct 2;16:8787. doi: 10.1038/s41467-025-63842-z (PMC12491539; doi:10.1038/s41467-025-63842-z)
Supplement: Supplementary file 20 — Reporting Summary [file 41467_2025_63842_MOESM20_ESM.pdf]

nature portfolio

Corresponding author(s): Shama Virani, Tom Dudding

Last updated by author(s): Aug 7, 2025

Reporting Summary

Nature Portfolio wishes to improve the reproducibility of the work that we publish. This form provides structure for consistency and transparency in reporting. For further information on Nature Portfolio policies, see our Editorial Policies and the Editorial Policy Checklist.

Statistics

For all statistical analyses, confirm that the following items are present in the figure legend, table legend, main text, or Methods section.

n/a	Confirmed	
☐	☒	The exact sample size (*n*) for each experimental group/condition, given as a discrete number and unit of measurement
☒	☐	A statement on whether measurements were taken from distinct samples or whether the same sample was measured repeatedly
☐	☒	The statistical test(s) used AND whether they are one- or two-sided<br>*Only common tests should be described solely by name; describe more complex techniques in the Methods section.*
☐	☒	A description of all covariates tested
☐	☒	A description of any assumptions or corrections, such as tests of normality and adjustment for multiple comparisons
☐	☒	A full description of the statistical parameters including central tendency (e.g. means) or other basic estimates (e.g. regression coefficient) AND variation (e.g. standard deviation) or associated estimates of uncertainty (e.g. confidence intervals)
☐	☒	For null hypothesis testing, the test statistic (e.g. *F*, *t*, *r*) with confidence intervals, effect sizes, degrees of freedom and *P* value noted<br>*Give P values as exact values whenever suitable.*
☐	☒	For Bayesian analysis, information on the choice of priors and Markov chain Monte Carlo settings
☒	☐	For hierarchical and complex designs, identification of the appropriate level for tests and full reporting of outcomes
☐	☒	Estimates of effect sizes (e.g. Cohen's *d*, Pearson's *r*), indicating how they were calculated

Our web collection on statistics for biologists contains articles on many of the points above.

Software and code

Policy information about availability of computer code

Data collection	Publicly available data was collected from dbGaP, or relevant data access protocols from UKBiobank and ALSPAC. Additionally, 10,000 samples were genotyped through the Center for Inherited Diseases (CIDR, X01HG010743) on the AllofUs array. Data from this genotyping has now been deposited on dbGaP.<br>For genome-wide association study (GWAS) analyses, genotype data from all arrays were converted to genome build GRCh38 using the LiftOver tool (https://genome.ucsc.edu/cgi-bin/hgLiftOver), allowing conversion from earlier genome builds. Imputation was performed separately for each array using the TOPMed imputation panel on the TOPMed Imputation Server (https://imputation.biodatacatalyst.nhlbi.nih.gov/). For HLA fine-mapping, variants directly genotyped on chromosome 6 were extracted, standardized to hg19 with LiftOver, and subsequently imputed. The imputation for SNPs and classical HLA class I and II alleles within the HLA region (Chromosome 6: 28Mb-34Mb) was carried out using the Michigan Imputation Server and the latest HLA Multi-ethnic reference panel (Four-digit Multi-ethnic HLA v2) (https://imputationserver.sph.umich.edu/#!).
Data analysis	Data analysis was conducted using a variety of open-source tools and specialized software. LiftOver to hg38 coordinates was performed on the Michigan Imputation Server using the TOPMed reference panel. Phasing was completed as a pre-imputation step with Eagle v2.4 on the Michigan Imputation Server, and Minimac4 was used for imputation.<br>Genotype data manipulation, quality control, and association analyses were carried out using PLINK versions 1.9 and 2.0. ADMIXTURE v1.3 was used for for maximum likelihood estimation of individual ancestries. Meta-analyses for non-HLA GWAS were conducted with METAL (version 2011–03-25).<br>R v4.1.2 was used for analyses such as Cochran's Q test, colocalisation of GWAS and eQTL mapping. Additionally, R was employed to generate plots and visualizations, including ADMIXTURE plots, circos Manhattan plots, and other graphical representations. Regional association plots were created with LocusZoom, and the Python-package forestplot: v0.4.0, pandas: v2.1.4, numpy: v1.26.3, matplotlib: v3.8.2 were used to create forest plot.

For HLA analaysis, PyMOL(TM) Molecular Graphics System, Version 2.5.0 was used to visualize protein structure in Figure 5 and 6. The R-package haplo.stats v.1.9.5.1 was used to predict haplotype associated to cancer risk. The R-package ggplot2: v.5.1, ggrepel: v0.9.6, dplyr: v1.1.4 were used to create Manhattan plot. Plink v1.9 was used to test logistic regression and meta-analyses of the variants and the outcome. bcftools was used to manage VCF files. Python script "liftOverPlink.py" from https://github.com/sritchie73/liftOverPlink was used to lift over the raw genotype data.
Schematic figures were created using BioRender (https://www.biorender.com/), and workflow diagrams for GWAS and HLA fine-mapping processes were designed in Miro (https://miro.com/app/dashboard/).

For manuscripts utilizing custom algorithms or software that are central to the research but not yet described in published literature, software must be made available to editors and reviewers. We strongly encourage code deposition in a community repository (e.g. GitHub). See the Nature Portfolio guidelines for submitting code & software for further information.

Data

Policy information about availability of data

All manuscripts must include a data availability statement. This statement should provide the following information, where applicable:

- Accession codes, unique identifiers, or web links for publicly available datasets
- A description of any restrictions on data availability
- For clinical datasets or third party data, please ensure that the statement adheres to our policy

The full GWAS summary statistics have been deposited in the MRC IEU OpenGWAS database and will be publicly available at https://gwas.mrcieu.ac.uk/, under accession numbers ieu-b-5129 (head and neck cancer), ieu-b-5130 (hypopharyngeal cancer), ieu-b-5131 (laryngeal cancer), ieu-b-5132 (oral cavity cancer), ieu-b-5133 (HPV-negative oropharyngeal cancer) and ieu-b-5134 (HPV-positive oropharyngeal cancer).
Additional datasets analyzed in this study are accessible through dbGaP (https://www.ncbi.nlm.nih.gov/gap/) as follows:
OncoArray Consortium - Lung Cancer Studies (dbGaP Study Accession: phs001273.v4.p2),
OncoArray: Oral and Pharynx Cancer (dbGaP Study Accession: phs001202.v2.p1),
National Cancer Institute (NCI) Head and Neck Cancer Study conducted on the HumanOmniExpress-12v1.0 array (dbGaP Study Accession: phs001173.v1.p1),
Genome-Wide Association Study of Oral Cavity, Pharynx, and Larynx Cancers in European, North, and South American populations (dbGaP Study Accession: phs002503.v1.p1).
Data from the UK Biobank and ALSPAC consortium, are available through their respective access protocols.

Research involving human participants, their data, or biological material

Policy information about studies with human participants or human data. See also policy information about sex, gender (identity/presentation), and sexual orientation and race, ethnicity and racism.

Reporting on sex and gender	Sex was genetically determined using genotype data, this sex variable was used throughout the study to account for potential genetic effects of sex.
Reporting on race, ethnicity, or other socially relevant groupings	In our study we used supervised ADMIXTURE analysis using 1000Genome as the reference to estimate genetic ancestry of the individuals. Geographic region was used to describe populations.
Population characteristics	Our study comprises approximately 19,073 cases of head and neck cancer, including 898 hypopharyngeal cancers, 4,409 laryngeal cancers, 5,596 oral cavity cancers, 1,473 HPV-negative oropharyngeal cancers, 2,212 HPV-positive oropharyngeal cancers, 1,726 oropharyngeal cancers with unknown HPV status, and 2,759 cases with unknown primary sites or overlapping diagnoses. Individuals were recruited from North America, South America, Europe, the Middle East, and South Asia. Models were adjusted for patient sex and stratified by age, geographic region, smoking status, drinking status, and subsite
Recruitment	All cases and controls were recruited from hospitals and research institutes. Cases were selected according to internationally accepted classifications of head and neck cancer.
Ethics oversight	For this meta-analysis and secondary analysis of individual-level data, ethics approval was granted by the International Agency for Research on Cancer (IARC) Ethics Committee (IEC 19-38).

Note that full information on the approval of the study protocol must also be provided in the manuscript.

Field-specific reporting

Please select the one below that is the best fit for your research. If you are not sure, read the appropriate sections before making your selection.

☒ Life sciences ☐ Behavioural & social sciences ☐ Ecological, evolutionary & environmental sciences

For a reference copy of the document with all sections, see nature.com/documents/nr-reporting-summary-flat.pdf

Life sciences study design

All studies must disclose on these points even when the disclosure is negative.

Sample size	No sample size calculation was performed, we included all available public data and our own generated data

Data exclusions	SNPs and individuals were removed at different quality control stages due to low-quality genotyping, high levels of missing genotyping data, or other relevant criteria, as detailed in our methods.
Replication	Rather than reduce sample size and power for separate discovery and replication cohorts, we consciously used all available data to power our study to identify novel loci at potentially lower allele frequencies. Due to our approach, we confirmed previously identified genomic loci and additionally identified new risk loci, some of which were rare but detectable with our sample size.
Randomization	No randomization was applied in this study. Head and neck cancer cases were categorized based on ICD codes corresponding to different cancer subsites, while controls were drawn from an unscreened, cancer-free population.
Blinding	Data was received with unique identifiers for which only the local study PIs could link directly to patients. For this analysis, we assigned another layer of unique identifiers such that samples could not be linked across other analyses that used this data with the original unique identifiers.

Reporting for specific materials, systems and methods

We require information from authors about some types of materials, experimental systems and methods used in many studies. Here, indicate whether each material, system or method listed is relevant to your study. If you are not sure if a list item applies to your research, read the appropriate section before selecting a response.

Materials & experimental systems

n/a	Involved in the study
☒	☐ Antibodies
☒	☐ Eukaryotic cell lines
☒	☐ Palaeontology and archaeology
☒	☐ Animals and other organisms
☒	☐ Clinical data
☒	☐ Dual use research of concern
☒	☐ Plants

Methods

n/a	Involved in the study
☒	☐ ChIP-seq
☒	☐ Flow cytometry
☒	☐ MRI-based neuroimaging

Plants

Seed stocks	*Report on the source of all seed stocks or other plant material used. If applicable, state the seed stock centre and catalogue number. If plant specimens were collected from the field, describe the collection location, date and sampling procedures.*
Novel plant genotypes	*Describe the methods by which all novel plant genotypes were produced. This includes those generated by transgenic approaches, gene editing, chemical/radiation-based mutagenesis and hybridization. For transgenic lines, describe the transformation method, the number of independent lines analyzed and the generation upon which experiments were performed. For gene-edited lines, describe the editor used, the endogenous sequence targeted for editing, the targeting guide RNA sequence (if applicable) and how the editor was applied.*
Authentication	*Describe any authentication procedures for each seed stock used or novel genotype generated. Describe any experiments used to assess the effect of a mutation and, where applicable, how potential secondary effects (e.g. second site T-DNA insertions, mosiacism, off-target gene editing) were examined.*
